# Early EGFR Inhibition Triggers Actionable Tumor and Microenvironmental Remodeling in Lung Cancer

**DOI:** 10.1158/2767-9764.CRC-26-0073

**Published:** 2026-08-26

**Authors:** Aaron C. Tan, Jia Chi Yeo, Jacob J.S. Alvarez, Mengyuan Pang, Yu Amanda Guo, Regina Hoo, Angela M. Takano, Boon-Hean Ong, Denise Goh, Joe Yeong, Zhengwei Wu, Ju Yuan, S. Shathishwaran, Ngak Leng Sim, Khi Pin Chua, Wai Leong Tam, Lan Ying Wang, Dawn P.X Lau, Stephanie P. Saw, Amit Jain, Gillianne G. Lai, Wan Ling Tan, Mei Kim Ang, Quan Sing Ng, Ravindran Kanesvaran, Darren W. Lim, Anders J. Skanderup, Daniel S. Tan

**Affiliations:** 1 Division of Medical Oncology, National Cancer Centre Singapore, Singapore, Singapore.; 2 Duke-NUS Medical School, National University of Singapore, Singapore, Singapore.; 3 Genome Institute of Singapore (GIS), Agency for Science, Technology and Research (A*STAR), Singapore, Singapore.; 4 Department of Computer Science, National University of Singapore, Singapore, Singapore.; 5 Centre for Biomedical Data Science & Programme in Cancer & Stem Cell Biology, Duke-NUS Medical School, National University of Singapore, Singapore, Singapore.; 6 Division of Pathology, Singapore General Hospital, Singapore, Singapore.; 7 Department of Cardiothoracic Surgery, National Heart Centre Singapore, Singapore, Singapore.; 8 Institute of Molecular and Cell Biology (IMCB), Agency for Science, Technology and Research (A*STAR), Singapore, Singapore.; 9 Cancer Science Institute of Singapore, National University of Singapore, Singapore, Singapore.; 10 Department of Biochemistry, Yong Loo Lin School of Medicine, National University of Singapore, Singapore, Singapore.

## Abstract

**Purpose:**

Despite widespread use of targeted therapies, little is known about how tumors and their microenvironment respond in the earliest phases of treatment and whether these early changes reveal new therapeutic opportunities. In this study, we investigated how short-term epidermal growth factor receptor (EGFR) inhibition reshapes tumor biology and the tumor microenvironment using comprehensive multiregion and spatial molecular profiling.

**Patients and Methods:**

Patients with stage IA–IIIA *EGFR*-mutated non–small cell lung cancer (NSCLC) received at least 4 weeks of gefitinib (250 mg daily) before surgery. Multiregion whole-exome and RNA sequencing of resected tumors were compared with a purity-matched treatment-naïve cohort.

**Results:**

Thirteen patients received neoadjuvant gefitinib for a median of 1.4 months, achieving objective response and disease control rates of 62% and 100%, respectively. Pathologic downstaging occurred in 46% of patients, with major pathologic response in 8%. Deep genomic and transcriptomic profiling revealed rapid tumor and microenvironmental adaptation to EGFR inhibition without emergence of common acquired resistance mutations such as *EGFR* T790M. Compared with treatment-naïve tumors, on-treatment tumors showed reduced tumor purity, decreased *EGFR* amplification, increased immunoregulatory and inflammatory gene expression, and a metabolic shift from glycolysis to oxidative phosphorylation, spatially validated.

**Conclusions:**

These findings show that early EGFR inhibition triggers coordinated remodeling of tumor cells and the microenvironment, uncovering new therapeutic vulnerabilities and opening a window for rational combination strategies to improve outcomes in *EGFR*-mutated NSCLC.

**Significance:**

This study demonstrated that neoadjuvant gefitinib is safe, feasible, and effective. Multiregion and spatial genomic and transcriptomic sequencing provided unique insight into adaptive response in the tumor and microenvironment. This window-of-opportunity study uncovered the potential for rational combination approaches to improve therapeutic efficacy in *EGFR*-mutated NSCLC.

## Introduction

First, second- and third-generation epidermal growth factor receptor (EGFR) tyrosine kinase inhibitors (TKI) are well-established first-line therapies in advanced or metastatic *EGFR*-mutated non–small cell lung cancer (NSCLC; ref. 1). There are also emerging data on combination approaches to first-line therapy to improve progression-free survival (PFS), combining EGFR TKIs with platinum-based chemotherapy (2) and amivantamab (3). In the early-stage setting, the randomized phase III ADAURA trial demonstrated improved overall survival (OS) with adjuvant osimertinib compared with placebo in resected stages IB to IIIA *EGFR*-mutated NSCLC (4).

In contrast, our understanding of the potential role for EGFR TKIs in the neoadjuvant setting remains nascent. Clinical benefits of the neoadjuvant approach include improved patient fitness and compliance and the unique opportunity to study *in vivo* response for prognostication and tailor adjuvant therapeutic options (5). Neoadjuvant immune checkpoint inhibition with nivolumab plus chemotherapy has demonstrated significant rates of pathologic complete response (pCR) and improved disease-free survival (DFS) compared with chemotherapy alone in stages IB to IIIA *EGFR*/*ALK* wild-type NSCLC (6). Early trials of neoadjuvant EGFR TKIs, however, did not selectively enroll for patients with activating *EGFR* mutations (7, 8). More recent studies of neoadjuvant osimertinib in resectable *EGFR*-mutated NSCLC have demonstrated modest pCR rates of 0% to 3.6% and major pathologic response (MPR) rates of 10.7% to 24% (9–11).

Crucially, the neoadjuvant approach also provides significant opportunities to obtain translational insights on the dynamics and mechanisms of antitumor response to EGFR inhibition (5). Despite high response rates to EGFR TKIs in advanced *EGFR*-mutated disease, the determinants of this therapeutic response remain poorly understood. Evaluating tumors at the time point of residual disease or interrogating potential drug-tolerant persister (DTP) cells provides a unique opportunity to explore the biology of drug resistance/tolerance (10). Uncovering novel targets can facilitate development of rational combination approaches to ultimately improve patient outcomes. Prior studies have implicated increased WNT/β-catenin activity in residual disease from a limited number of neoadjuvant osimertinib-treated tumors (9). Studies of patient-derived xenografts and cell lines have implicated upregulation of YAP and the NF-κB transcription factor RELA in DTP cells (12, 13). However, comprehensive multi-omics sequencing studies of EGFR TKI–treated tumors, particularly multiregion studies to account for intratumoral heterogeneity, have not been conducted previously.

We conducted a window-of-opportunity study to investigate the safety, feasibility, and efficacy of neoadjuvant gefitinib treatment prior to surgery for resectable *EGFR*-mutated NSCLC. Comprehensive in-depth multiregion tissue sequencing was conducted to identify early dynamic alterations in *EGFR*-mutated NSCLC tumor biology in response to EGFR TKI therapy. In particular, our study aimed to uncover markers of EGFR TKI sensitivity or resistance that may help guide subsequent therapeutic options.

## Patients and Methods

### Study design and participants

This single-center, open-label, phase II trial enrolled patients between February 2015 and March 2020 at the National Cancer Centre Singapore. Adult patients with histologically confirmed *EGFR* mutation–positive stages IA to IIIA NSCLC deemed surgically resectable were eligible. Other major inclusion criteria included a primary tumor at least 2 cm (T1b) in size and patients assessed as fit for surgery with either lobectomy/pneumonectomy with or without lymph node sampling. Key exclusion criteria included the presence of *EGFR* exon 20 insertions, *EGFR* T790M mutations, prior exposure to radiotherapy or systemic anticancer therapy for NSCLC, interstitial lung disease or pulmonary fibrosis, and any other serious concomitant systemic disorder or laboratory finding that would compromise the patient’s ability to complete the study or contraindicate the use of gefitinib. The protocol was approved by the SingHealth Centralized Institutional Review Board (#2018/2722), and all patients provided written informed signed consent. This study was conducted in accordance with the ethical principles of the Declaration of Helsinki, the principles of Good Clinical Practice, and all applicable regulatory requirements. This study is reported in accordance with the Consolidated Standards of Reporting Trials (CONSORT) guidelines.

### Procedures

**Figure 1.**
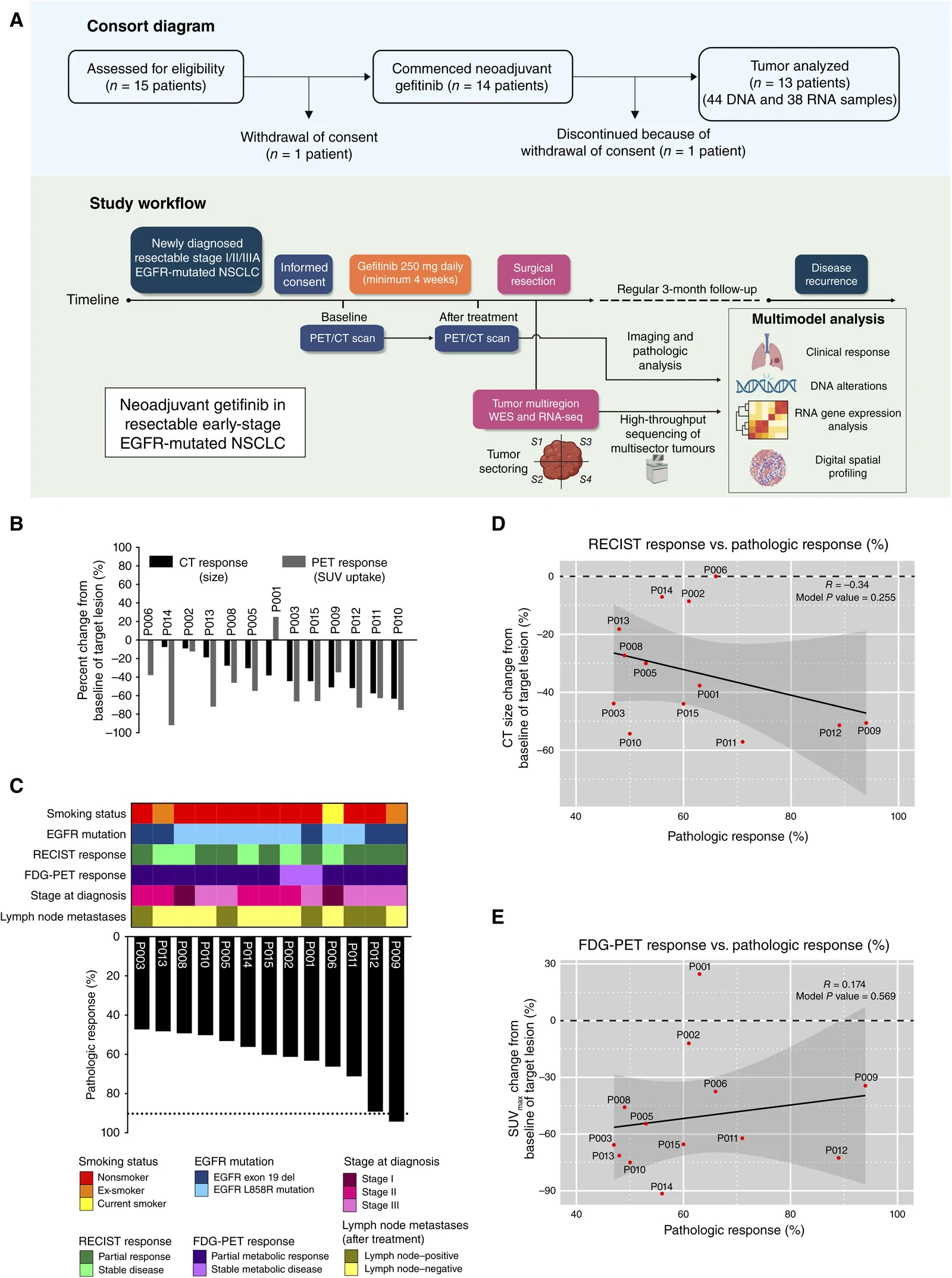
Clinical assessments of response and outcomes to neoadjuvant gefitinib-treated *EGFR*-mutated NSCLC. **A,** Consort diagram and study workflow. Study includes the baseline PET/CT scan and treatment with gefitinib 250 mg daily for a minimum of 4 weeks, followed by posttreatment PET/CT scan and surgical resection. Multiregion WES and RNA-seq were then performed on tumor sectors for multi-omics analysis. **B,** Waterfall plot. Dotted line indicates PR or partial metabolic response (PMR; <30% change in baseline). **C,** Pathologic assessment of response. Dotted line indicates the threshold for a MPR (<10% viable tumor). **D** and **E,** Correlations of clinical measures of response. **D,** CT response compared with pathologic response. **E,** PET response compared with pathologic response.

All patients had baseline tumor staging, including 2[18F]fluoro-2-deoxy-D-glucose (FDG)-PET/CT scans and contrast-enhanced CT or MRI of the brain (Fig. 1A). Mediastinal nodal evaluation, if indicated, was performed by bronchoscopy. Eligible patients then received a minimum of 4 weeks of oral gefitinib 250 mg daily prior to surgery. Treatment was continued until the day of surgery in the absence of disease progression, occurrence of unacceptable toxicity, or withdrawal of patient consent. Dose interruptions of up to 2 weeks were allowed for recovery from clinically significant adverse events (AE). Treatment was permanently discontinued in the event of AEs for which discontinuation was considered necessary by the investigator, dose delays of more than 2 weeks, and noncompliance with the administration of gefitinib. All patients were offered conventional adjuvant therapy as clinically indicated, including radiotherapy or systemic chemotherapy, as per institutional standards of care.

### Study assessments

Disease status was assessed by investigators according to Response Evaluation Criteria in Solid Tumors (RECIST) v1.1 (14) at baseline and prior to surgery after a minimum 4 weeks of gefitinib, using FDG-PET and contrast-enhanced CT scans. AEs were monitored throughout the study and graded according to the National Cancer Institute Common Terminology Criteria for Adverse Events v4.0. Laboratory monitoring for toxicity was conducted at baseline, at 2 weeks, at the time of surgery, and more frequently as required at the investigator’s discretion. Postoperative follow-up was scheduled every 3 months for the first 2 years, followed by six monthly for the next 3 years and yearly thereafter. CT scans were performed to follow-up disease status at regular intervals decided by the investigator according to local standards of care.

Resected tumors were sectioned horizontally, and tumor tissue in each section was cut into four quadrants as described previously (15). Pathologic staging of the primary lung tumor and lymph node surgical specimens was conducted according to the criteria of the American Joint Committee on Cancer (seventh edition). Pathologic response was assessed using previously described criteria (16) in which tumors with ≤10% of residual viable tumor cells are considered to have had a MPR. In addition to routine standard histopathologic assessment, sectors with reasonably high tumor content were snap-frozen for DNA/RNA sequencing (RNA-seq). Neighboring horizontal sections were used for histologic analyses, and adjacent normal lung tissue or blood was used as matched normal control.

### Multiregion whole-exome sequencing and RNA-seq

DNA and total RNA extractions were performed from frozen tissues using Qiagen AllPrep Universal Kit, and the DNA was subjected to library preparations for sequencing with a target coverage of 250× (median 269×; range, 215×–363×). Targeted amplicon deep sequencing, variant calling from exome sequencing, phylogenetic analysis, mutation signature analysis, copy-number analysis, and RNA-seq analysis are described in the following sections. A matched treatment-naïve cohort (*n* = 21) of patients with early-stage *EGFR*-mutated NSCLC who underwent surgical resection and multiregion whole-exome sequencing (WES)/RNA-seq was also curated for the comparison of genomic and transcriptomic features (bioRxiv 2023.07.04.547310). This matched treatment-naïve cohort was sequenced at a target coverage of 50× (median 47×; range, 20×–86×). For comparisons at similar sequencing depth, additional sequencing was done for the neoadjuvant gefitinib treatment to obtain a target coverage of 50× (median 66×; range, 24×–162×). Postsequencing quality control (QC) revealed that patient P014 had very low tumor purity, which compromised the reliability of variant calling and downstream analyses. Additionally, discordant mutation overlap between P014 tumor sectors indicated potential technical artifacts or insufficient data quality. As a result, patient P014’s DNA and RNA samples were excluded from our analysis to ensure data integrity. Somatic variant and copy-number variation (CNV) identification, tumor clonality, and CNV analysis are shown in Supplementary Methods.

### Tumor purity matching

To control for purity levels during analyses, subsets of each cohort were sampled without replacement from the bottom 40th percentile of purity and below. Only samples with both WES data and RNA-seq were used, and a maximum of three samples were to be from any patient to prevent overrepresentation. There was no significant difference in purity between the two resultant subsets. For analyses involving patient-level metrics such as number of clusters, all sectors were used for each patient selected in the purity-matched cohorts.

### RNA data analysis

RNA data were aligned using STAR aligner (17), and gene expression counts were quantified using RSEM (18). Lowly expressed genes were filtered out using the function “filterByExpr” (min.count = 10, min.prop = 0.05) in the “edgeR” R package (19). Preprocessed RSEM counts were next normalized using the weighted trimmed mean of M-values method and then transformed into log counts per million (log_2_-CPM) using the R function “voom” (20). Gene set enrichment analysis (GSEA) was carried out using the R package “fgsea” (bioRxiv 060012) with gene sets downloaded from the Molecular Signatures Database (MSigDB, v7.5). Linear regression was applied to the neoadjuvant gefitinib cohort in order to uncover genes that were significantly associated with the percentage of residual viable tumor cells.

For spatial transcriptomics, NanoString GeoMx raw gene expression count files were preprocessed using the *standR* package (21). In brief, a *SpatialExperiment* object with count level was constructed to include all region-of-interest (ROI) information, raw counts, and log_2_-CPM. Gene-level and ROI-level QC was performed to exclude genes with low expression values in more than 90% of the ROIs and ROIs with less than 100 cell count range prior to downstream analysis. After QC processing, we used the *geomxBatchCorrection* function for RUV4 batch correction and normalization with parameter k = 1. Only “tumor”- and “border”-labeled ROIs were subset for further analysis. We next performed differential expression analysis with the *limma-voom* and *edgeR* packages. Weight matrices generated from the RUV4 and treatment groups (treatment-naïve vs. neoadjuvant gefitinib) were included in the *limma* linear model matrix as covariates. GSEA was performed using the *fgsea* package with gene set h.all.v2023.1.Hs.symbols.

### Immune cell estimation and activity scoring

Assays of immune cell populations were estimated using CIBERSORTx algorithm (22). T cell–inflamed gene expression profile (GEP) score was determined by the weighted sum of normalized log_2_-CPM expression using the weights derived from Cristescu and colleagues (23). The tumor inflammation status clustering (R package CancerSubtype, function ExecuteCC, k = 2, repeats *n* = 5,000) was defined and calculated as previously described (24).

### Immunofluorescence staining and analyses

Formalin-fixed, paraffin-embedded neoadjuvant gefitinib tumor samples were obtained and cut into 4-μm sections. The sections were deparaffinized, and antigen retrieval was carried out in citrate buffer in the presence of 0.5% Tween-20. The sections were further permeabilized in 0.2% Triton X-100 and then quenched with TruBlack (Gold Biotechnology). The sections were then incubated with anti–phosphofructokinase 1 (PFK1; sc-166722) from Santa Cruz, anti–pan cytokeratin eFluor 570 (41-9003-82) from Invitrogen, and anti-ATPB (ab170947) from Abcam. Alexa Fluor 488 donkey anti-rabbit (A21206) antibody from Life Technologies and Alexa Fluor 647 goat anti-mouse (A28181) antibody from Thermo Fisher Scientific were used as secondary antibodies. Images were visualized and captured using a Zeiss Axio Observer 3 epifluorescence microscope, and quantifications of staining intensity and staining co-localizations were performed using ImageJ.

### Study outcomes

The primary endpoint was the detection of biomarkers for TKI sensitivity in responders versus nonresponders, as categorized by RECIST v1.1 tumor evaluation or median percentage tumor shrinkage. Secondary endpoints included safety, feasibility, and efficacy as determined by objective response rate (ORR) after neoadjuvant gefitinib as evaluated by RECIST v1.1. Clinical exploratory endpoints included the proportion of patients downstaged after neoadjuvant gefitinib and long-term outcomes as measured by DFS. Translational exploratory endpoints included the evaluation of the clonal heterogeneity using WES and RNA-seq.

### Statistical considerations

Descriptive statistics were used to summarize patient characteristics, safety, and efficacy data. DFS was calculated from the date of surgery until disease recurrence or death. DFS and OS were estimated by the Kaplan–Meier method. Reported *P* values are two-sided, with significance level set at 0.05 for all analyses unless otherwise noted.

## Results

### Patient characteristics

From February 2015 to November 2018, 14 patients were enrolled and received at least one dose of neoadjuvant gefitinib (Fig. 1A). One patient discontinued gefitinib treatment and withdrew from the study prior to surgery without experiencing toxicity or disease progression. The baseline demographics of the remaining 13 patients on trial are as summarized in Table 1. The median age was 60 years (range, 48–78 years), with patients being nearly equally split between genders (seven females vs. six males), as well as *EGFR* mutation type (six with *EGFR* exon 19 deletion vs. seven with *EGFR* L858R mutation). Approximately half of patients had stage IIIA NSCLC (*n* = 6, 46%), with a majority of them being never-smokers (*n* = 10, 77%).

**Table 1.** Patient characteristics.

Characteristics (*n* = 13)	*n* (%)
Age – years, median (range)	60 (48–78)
Sex	​
Female	7 (54)
Male	6 (46)
EGFR mutation^a^	​
Exon 19 deletion	6 (46)
L858R mutation^b^	7 (54)
Clinical stage at diagnosis	​
I	2 (15)
II	5 (38)
IIIA	6 (46)
Smoking status	​
Never	10 (77)
Former or current	3 (23)

a One patient had a coexisting ROS1 rearrangement.

b One patient with exon 21 L858R and V834L comutation.

### Neoadjuvant gefitinib demonstrates favorable safety and surgical feasibility

The median time on treatment was 41 days (range, 24–56 days), with no unexpected toxicities or AEs arising from neoadjuvant gefitinib treatment (Supplementary Table S1). One (8%) patient ceased therapy because of toxicity with G3 aspartate aminotransferase/alanine aminotransferase elevation that resolved with the discontinuation of gefitinib and subsequently underwent an uncomplicated surgical resection. There were no other G3/4 AEs, with the most common G1/2 AEs reported being rash (*n* = 7, 54%), diarrhea (*n* = 4, 31%), and oral ulcers (*n* = 1, 15%; Supplementary Table S1). Almost all patients (*n* = 12, 92%) underwent surgical resection as planned with no treatment-related surgical delays (median time to surgery = 1.6 months; range, 0.9–4.4 months). One patient elected to defer surgery to 4.4 months after neoadjuvant gefitinib.

### Neoadjuvant gefitinib elicits promising clinical and pathologic responses

After neoadjuvant gefitinib, eight (62%) patients had partial response (PR) and five (38%) patients had stable disease (SD) for an ORR of 62% and disease control rate of 100% (Fig. 1B). Upon FDG-PET imaging, 11 (85%) patients had partial metabolic response and two (15%) patients had stable metabolic disease (Fig. 1B). Pathologic findings at time of resection revealed pathologic downstaging in six (46%) patients, with one (8%) patient having T-stage upstaging (T2–T4) due to a finding of intrapulmonary lymphovascular micrometastases (Supplementary Table S2; Supplementary Fig. S23). All patients had an R0 resection status, with four of nine (44%) patients with node-positive disease at baseline reporting a nodal pCR (Supplementary Table S2).

After surgical resection, seven (54%) patients went on to receive adjuvant systemic therapy of either platinum-doublet chemotherapy (*n* = 6, 46%) or EGFR TKI therapy (*n* = 1, 8%), of which one received platinum-doublet chemotherapy followed by concurrent chemoradiation. With a median follow-up of 38.1 months (range, 4.8–54.8 months), seven (54%) patients recurred with a median DFS of 20.2 months (Supplementary Fig. S1A). Among these patients with disease recurrence, five proceeded to receive palliative EGFR TKI therapy, with three having a PR, one with progressive disease, although with leptomeningeal only recurrence, and one being lost to follow-up. The median OS for this study was 53.5 months (Supplementary Fig. S1B).

MPR (<10% residual viable tumor cells) occurred in one (8%) patient (P009), with near-MPR found in another patient (P012; 11% residual viable tumor cells; Fig. 1C). In a patient (P002) with SD on follow-up CT, pathologic findings demonstrated 39% residual viable tumor cells and T-stage downstaging from T2a to T1a (35 to 20 mm). We did not, however, observe any significant correlation at the study cohort level between RECIST response (R = −0.34, *P* = 0.255) or FDG-PET response (R = 0.174, *P* = 0.569) against pathologic response (Fig. 1D and E). This indicates that discordance may be observed between different modalities and measures of treatment response.

### Early genomic tumor adaptations following neoadjuvant gefitinib

Multiregion WES was next performed to compare the genomic changes between neoadjuvant gefitinib (269× WES, 12 patients, *n* = 44 samples) and against a control treatment-naïve NSCLC cohort (50× WES, 17 patients, *n* = 66 samples; Supplementary Table S3).

**Figure 2.**
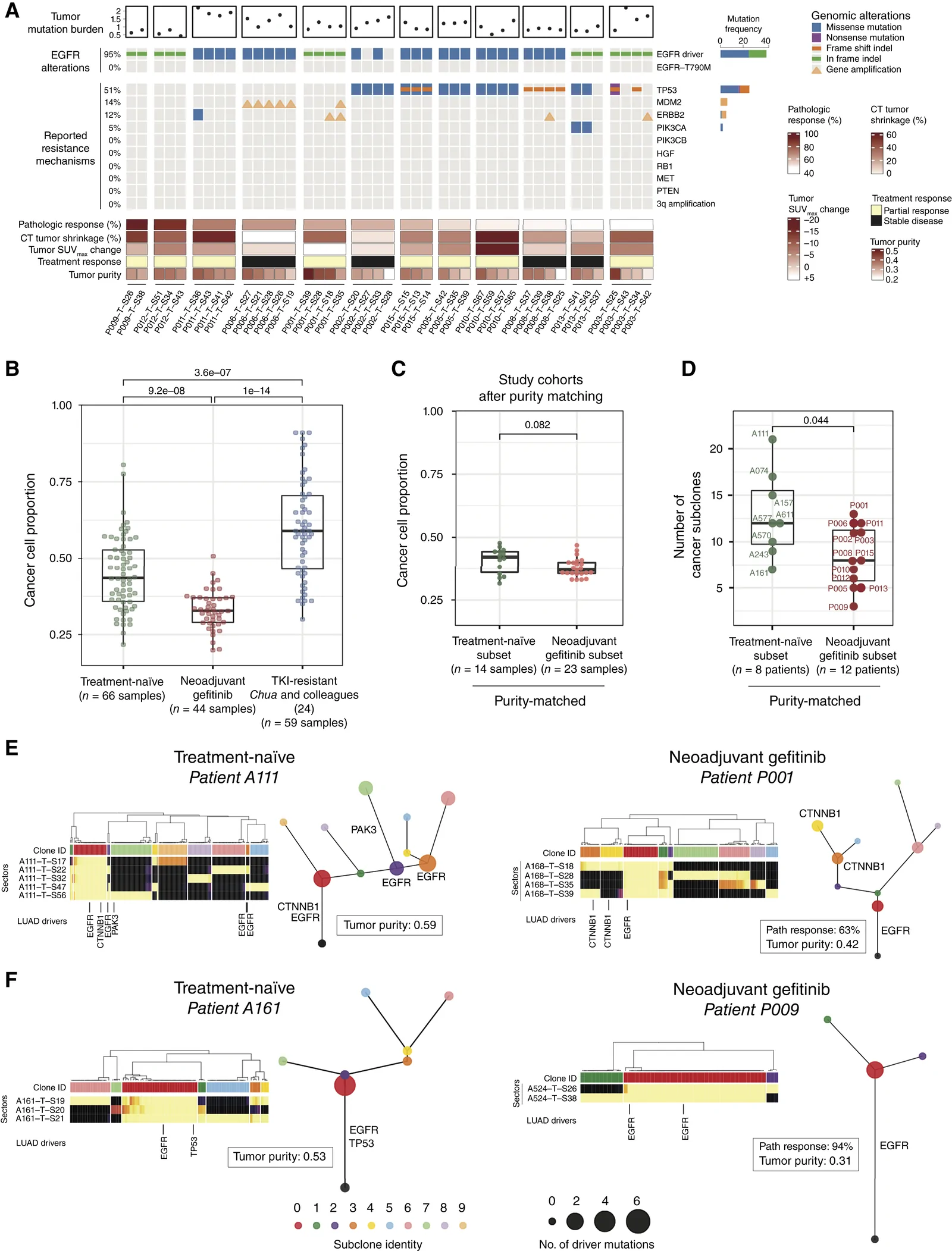
Genomic landscape of neoadjuvant gefitinib-treated *EGFR*-mutated NSCLC tumors. **A,** Oncoprint of *EGFR* alterations and reported EGFR TKI resistance genes within the neoadjuvant gefitinib patient cohort. **B,** Estimated cancer cell proportion distributions of different NSCLC cohorts. Tumors from the TKI-resistant group comprises patients from the study by Chua and colleagues (24). **C,** Cancer cell proportions for a subset of the treatment-naïve and neoadjuvant gefitinib cohort, selected at random for downstream purity-matched analyses. **D,** Distribution of the number of cancer subclones estimated in patient-level analysis between purity-matched treatment-naïve and neoadjuvant gefitinib tumors at 50× and 250× depth WES respectively. **E,** Inferred phylogenetic structures of treatment-naïve (A111) and neoadjuvant gefitinib (P001) patients with NSCLC with the highest number of cancer subclones within their respective cohorts. **F,** Inferred phylogenetic structures of treatment-naïve (A161) and neoadjuvant gefitinib (P009) patients with NSCLC with the lowest number of cancer subclones within their respective cohorts. Only known lung adenocarcinoma (LUAD) and TKI-resistant (*n* = 96) genes are annotated for each clone on the tree, and the mutation burden of a given clone is represented by the relative distances between nodes.

Although all of our study samples had detectable *EGFR* mutations, neoadjuvant gefitinib tumors had no appreciable development of *EGFR* T790M gatekeeper or other genomic features of reported EGFR TKI resistance (24), aside from an observed increase in *ERBB2* gene amplification (Fig. 2A; Supplementary Fig. S2A and S2B; Supplementary Tables S4 and S5). However unlike EGFR TKI–resistant tumors, these *ERBB2* amplifications in neoadjuvant gefitinib samples were of low magnitude and were not overexpressed (Supplementary Fig. S3). Tumor mutation burden between the two study cohorts was also found to be not significantly different (Fig. 2A; Supplementary Fig. S2C). We then expanded our genomic analysis to identify novel non-EGFR TKI resistance genes and found that neoadjuvant gefitinib tumors had higher missense mutations in the *LRBA* and *PTPN23* genes (*P* = 0.0208, Supplementary Fig. S4). These mutations were, however, variants of unknown significance, have not yet been characterized in the context of *EGFR*-mutated NSCLC biology, and their functional implications have yet to be elucidated (Supplementary Table S4). We lastly evaluated co-occurring mutations in these *EGFR*-mutated neoadjuvant gefitinib tumors (*ERBB2*, *LRBA*, *MDM2*, *PIK3CA*, *PTPN23*, and *TP53*) for potential associations with treatment response. Only *TP53* mutations were significantly associated with poorer pathologic response (*P* = 0.0047, Supplementary Fig. S5), whereas no consistent associations were identified for the other genes. Given the limited cohort size, this finding will require validation in larger studies.

Taken together, this suggests that known resistance mechanisms to gefitinib may not commonly be present *de novo* or during the early phases of EGFR TKI therapy. Nevertheless, evidence of early tumor genomic adaptations toward EGFR TKIs was observed in our study.

First, neoadjuvant gefitinib tumors demonstrated lower tumor purity against treatment-naïve or EGFR TKI–resistant samples (Fig. 2B). This suggests a significant reduction in cancer cell populations upon gefitinib exposure, with a subsequent regrowth of cancer cells after development of EGFR TKI resistance. Importantly, given these significant cohort-level differences in cancer–stroma proportions and to enable the accurate molecular analysis of gefitinib perturbations, we next selected a subset of tumors with comparable tumor purities between the treatment-naïve and neoadjuvant gefitinib cohorts (Fig. 2C; neoadjuvant gefitinib: 12 patients, *n* = 23 samples vs. treatment-naïve: 9 patients, *n* = 14 samples; see “Patients and Methods” – “Tumor purity matching”).

Second, as multiregion sequencing enabled the estimation of each tumor’s clonal phylogenies, it was found that neoadjuvant gefitinib samples had a significantly lower number of tumor subclones and clonal diversity (25) when compared with purity-matched treatment-naïve patients (Fig. 2D; Supplementary Fig. S6A and S6B). This phenomenon was consistent even when controlling for WES sequencing depth (Supplementary Fig. S6C). However, through the construction of individual patient cancer mutation phylogenetic trees, we observed that the highest and lowest representative clone trees between treatment-naïve and neoadjuvant gefitinib-treated patients did not show overt qualitative differences (Fig. 2E; Supplementary Figs. S7 and S8). This suggests that the overall clonal architecture remained relatively stable despite neoadjuvant gefitinib treatment.

Third, it was discovered that neoadjuvant gefitinib tumors also showed fewer high-level *EGFR* amplification events compared with treatment-naïve samples (6.8% vs. 16.7% respectively; Supplementary Fig. S9A). Interestingly, in the EGFR TKI–resistant setting, low frequencies of *EGFR* amplifications were more commonly observed in *EGFR* T790M-negative tumors compared with *EGFR* T790M-positive tumors (9.5% vs. 21.1% respectively; Supplementary Fig. S9A). This indicates a possible role for *EGFR* amplification in the developmental trajectory of EGFR TKI resistance. Although tumors with *EGFR* gene amplification had elevated copy numbers of the mutant *EGFR* alleles, we did not find a significant difference in *EGFR* expression levels between *EGFR*-amplified versus -neutral tumors within the neoadjuvant gefitinib tumors (Supplementary Fig. S9B and S9C).

It should, however, be highlighted that these observed genomic alterations do not correlate with any clinical patient response metrics such as CT, PET, or pathologic response (Supplementary Fig. S10), suggesting that these factors alone are insufficient to lead to and predict for recurrence and that additional oncogenic hits are required.

### Neodjuvant gefitinib remodels the tumor microenvironment by enhancing immune cell infiltration and inflammatory response

In addition to WES, RNA-seq was also conducted to ascertain the gene expression changes occurring within neoadjuvant gefitinib tumors (12 patients, *n* = 38 samples) against the control treatment-naïve group (18 patients, *n* = 47 samples; Supplementary Table S6).

**Figure 3.**
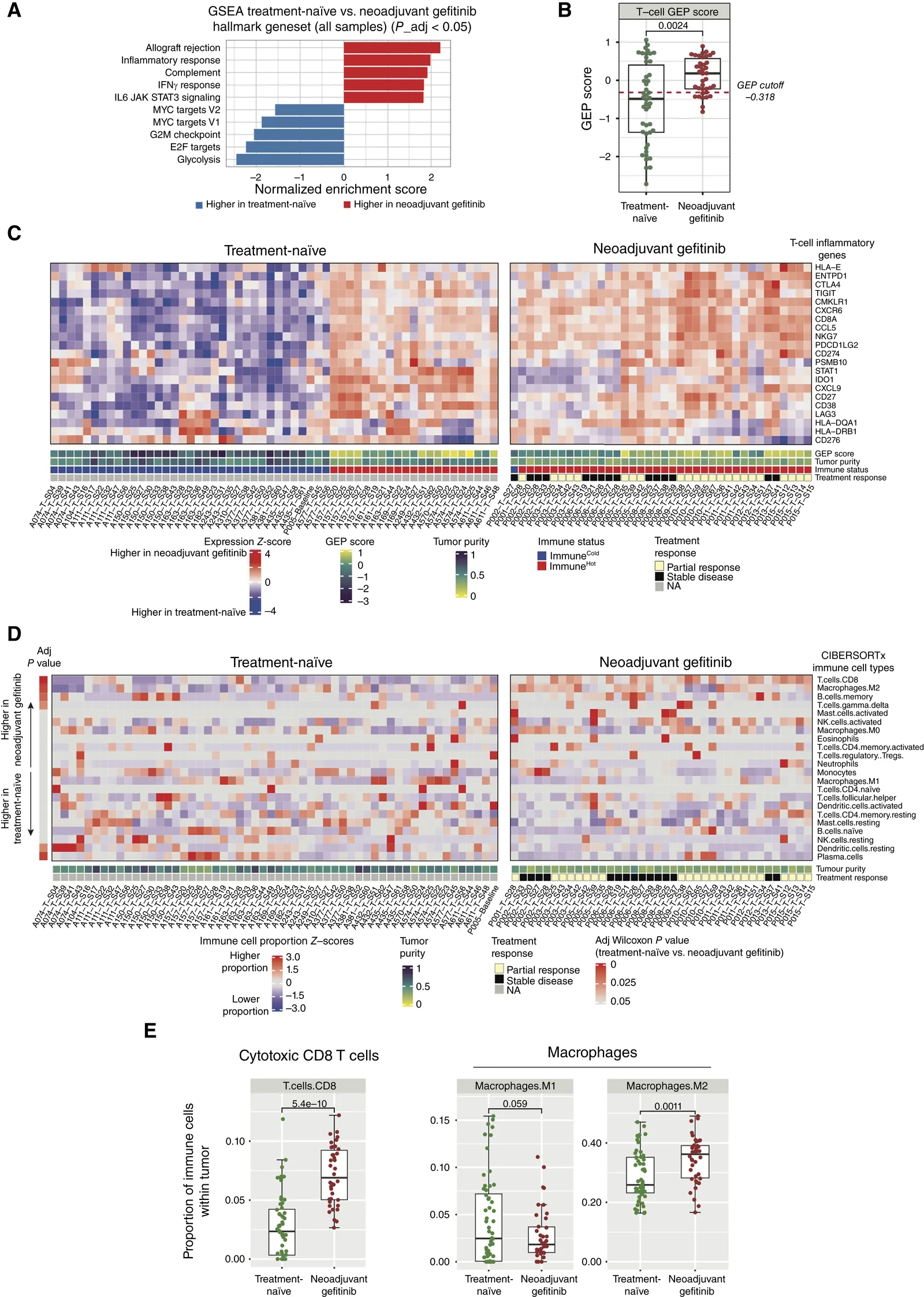
Transcriptomic analysis of neoadjuvant gefitinib-treated *EGFR*-mutated NSCLC tumors. **A,** Top 5 enriched mSigDB “hallmark” gene pathways found in neoadjuvant gefitinib or treatment-naïve tumors in GSEA. **B,** T-cell inflammation GEP scores between treatment-naïve and neoadjuvant gefitinib tumors. The horizontal line demarcates the GEP cutoff score (GEP = −0.318) as previously reported by Cristescu and colleagues (23). **C,** Heatmap summarizing the expression levels of key immune inflammatory genes within patient tumors, as well as their corresponding tumor classification into either immune^Hot^ or immune^Cold^ status. **D,** Heatmap of immune cell types estimated by CIBERSORTx ranked by the order and direction of significance (adjusted *P* value) between treatment-naïve vs. neoadjuvant gefitinib cohorts. **E,** Boxplots of selected CIBERSORTx cell-type proportions present within treatment-naïve vs. neoadjuvant gefitinib tumors.

As expected from the marked tumor shrinkage observed in the neoadjuvant gefitinib cohort (Fig. 1B and C), GSEA of these tumor RNA samples indicated the suppression of gene pathways associated with cell division, specifically “E2F targets,” “G2M checkpoint,” and “MYC targets V1” (Fig. 3A). Interestingly, our prior observation of reduced cancer cell populations in neoadjuvant gefitinib tumors was accompanied by a reciprocal increase in immune activity, as demonstrated by the enrichment of immunoregulatory and inflammatory-related terms in GSEA (Fig. 3A).

To further investigate this association, the T cell–inflamed GEP scores were evaluated. It was observed that neoadjuvant gefitinib samples had a significantly higher GEP score than treatment-naïve tumors (mean score; treatment-naïve = −0.58 vs. neoadjuvant gefitinib = 0.12, *P* = 0.0024; Fig. 3B). Additionally, tumor-level gene expression analysis also found high levels of key immunoregulatory and inflammatory genes in neoadjuvant gefinitib tumors, with almost the entire treatment cohort being classified as an immune^hot^ tumor (12/12 patients). In contrast, a majority of treatment-naïve patients were noninflamed, with only 39% found to be immune^hot^ (7/18 patients; Fig. 3C). Lastly, computational elucidation of the infiltrating immune cell subsets using CIBERSORTx (22) highlighted an increased presence of infiltrating immune cells in neoadjuvant gefitinib tumors; including cytotoxic CD8 T cells and M2 macrophages (Fig. 3D and E; Supplementary Fig. S11). To further characterize these infiltrating immune cells, we used whole-transcriptome digital spatial profiling (DSP), evaluating PanCK+ cancer regions, immune T cells (CD3^+^ regions), and macrophage (CD68^+^ regions) compartments. There was no difference in T-cell functional phenotype scores for cytotoxic versus exhausted T cells (Supplementary Fig. S24). However, there was a trend towards a higher proportion of M2 macrophages in neoadjuvant versus treatment-naïve tumors (Supplementary Fig. S25).

Taken together, our data reveals that during gefitinib treatment, *EGFR*-mutated tumors will undergo significant changes in the tumor microenvironment (TME), with infiltrating immune cells potentially playing a novel, but yet to be determined, role in EGFR TKI drug response.

**Figure 4.**
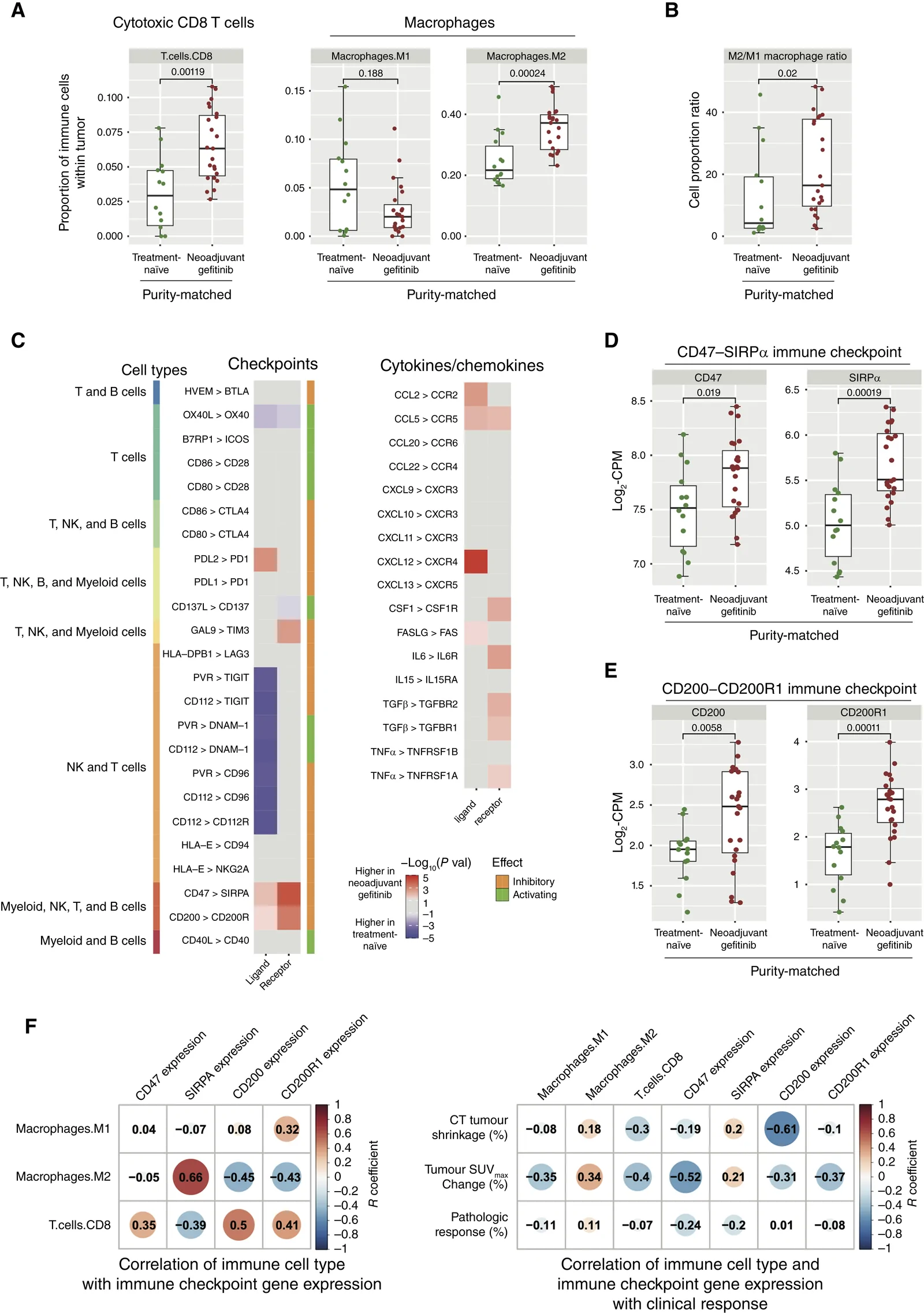
Analysis of immune checkpoint and cytokine/chemokine responses in purity-matched neoadjuvant gefitinib *EGFR*-mutated NSCLC tumors. **A** and **B,** Boxplots of selected CIBERSORTx cell-type proportions present within purity-matched treatment-naïve vs. neoadjuvant gefitinib tumors. **C,** Gene expression comparison of immune checkpoint and cytokine/chemokine ligand–receptor pairs. **D** and **E,** Boxplot of gene expression levels comparing CD47–SIRPA and CD200–CD200R1 immune checkpoint pairs in treatment-naïve vs. neoadjuvant gefitinib tumors. **F,** Correlation of immune cell type and immune checkpoint gene expression with clinical response, including CT tumor shrinkage, SUV_max_ change, and pathologic response.

To control for cancer–stroma-level differences between treatment cohorts and to better delineate EGFR TKI–induced immune and gene expression changes, we next repeated CIBERSORTx immune deconvolution within our purity-matched tumor cohort. Notably after purity-matching, the high levels of cytotoxic CD8 T cells and M2 macrophages continued to persist in neoadjuvant gefitinib tumors (Fig. 4A and B; Supplementary Figs. S12 and S13).

Considering the contrasting nature of these antitumorigenic CD8 T cells and protumorigenic M2 macrophages in cancer (26, 27), we hypothesized that the immune activity present in the neoadjuvant gefitinib-treated TME may be characterized by a dynamic equilibrium between tumor-suppressive and tumor-promoting forces. This was supported by our gene expression analysis whereby we found upregulation of immune tolerance promoting CD47–SIRPα (28) and CD200–CD200R1 (29) checkpoint genes; with increased levels of protumorigenic CCL5–CCR5 (30) and CXCL12 chemokine (31) axis genes in neoadjuvant gefitinib tumors (Fig. 4C–E). Additionally, although the downregulation of the protumorigenic PVR/PVRL2 ligands may help enhance immune activity during EGFR TKI therapy (Supplementary Fig. S14A and S14B), this advantage may likely be mitigated by the low expression of the antitumorigenic T cell–activating OX40L–OX40 pathway genes (Supplementary Fig. S14C; refs. 32, 33). Although there were significant negative correlation between CD47 expression with tumor SUV_max_ change (R = −0.52, *P* value = 0.01), as well as CD200 expression with CT tumor shrinkage (R = −0.608, *P* value = 0.002), these trends were not consistently observed across all cases (Fig. 4F; Supplementary Figs. S15 and S16). Lastly, comparison with EGFR TKI–resistant tumors (24) revealed the elevated expression of these CD47–SIRPα and CD200–CD200R1 pathways specifically only during the neoadjuvant gefitinib phase (Supplementary Fig. S17), highlighting a potential therapeutic window for combining gefitinib with immune checkpoint inhibitors in patients.

Overall, these findings hint at a complex interplay between immune cells and checkpoint pathways, underscoring the need for a deeper understanding and comprehensive strategies to target both tumor-suppressive and tumor-promoting immune components.

### Neoadjuvant gefitinib induces a metabolic shift from a glycolytic to oxidative phosphorylation state

**Figure 5.**
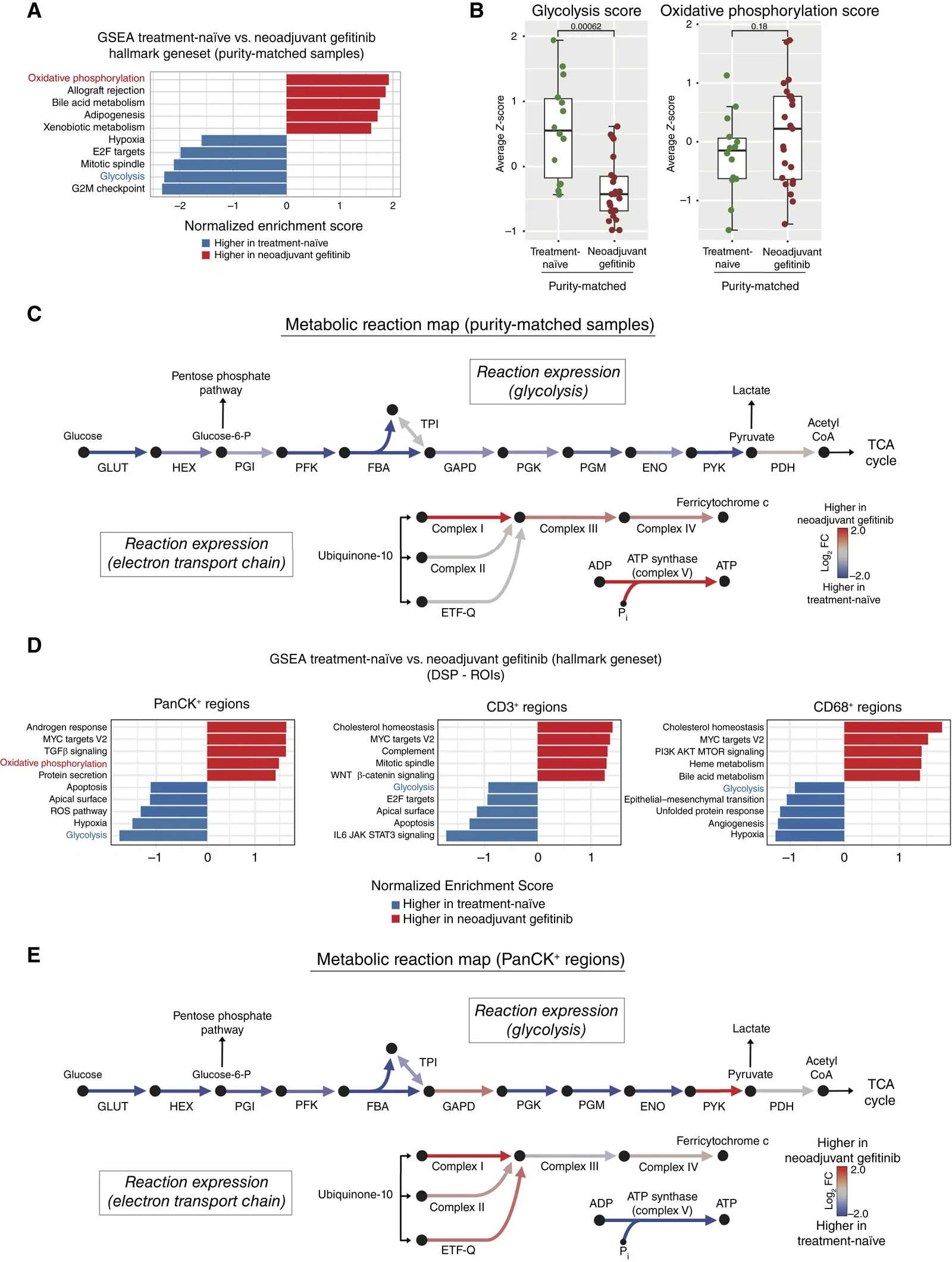
Purity-matched neoadjuvant gefitinib-treated *EGFR*-mutated NSCLC tumors undergo a glycolytic to oxidative phosphorylation metabolic switch. **A,** Top 5 enriched mSigDB “hallmark” gene pathways found in purity-matched neoadjuvant gefitinib or treatment-naïve tumors in GSEA. **B,** Average glycolytic or oxidative phosphorylation gene expression *Z*-scores in treatment-naïve vs. neoadjuvant gefitinib tumors. **C,** Purity-matched metabolic pathway map of differentially expressed glycolysis and oxidative phosphorylation reactions in treatment-naïve vs. neoadjuvant gefitinib tumors. **D,** GSEA of top 5 enriched mSigDB “hallmark” gene pathways found via DSP ROIs (PanCK+, CD3+, and CD68+ regions) in representative treatment-naïve vs. neoadjuvant gefitinib tumors. **E,** PanCK+ metabolic pathway map of differentially expressed glycolysis and oxidative phosphorylation reactions in representative treatment-naïve vs. neoadjuvant gefitinib tumors. FC, fold change; TCA, tricarboxylic acid.

We also performed GSEA on these purity-matched samples and found that “oxidative phosphorylation” and “glycolysis” gene sets were among the top ranked terms from the neoadjuvant gefitinib and treatment-naïve groups, respectively (Fig. 5A and B). Metabolic pathway plotting using Escher (34) revealed that the high glycolysis levels within treatment-naïve tumors were centered around several key reactions, including glucose transporter, PFK, fructose-bisphosphate aldolase, phosphoglycerate mutase, and pyruvate kinase. In contrast, neoadjuvant gefitinib tumors had elevated oxidative phosphorylation arising from high complexes I, III, and IV and ATP synthase levels (Fig. 5C). Notably, glycolytic or oxidative phosphorylation gene expression signatures showed no significant correlation with patient clinical features, indicating that these metabolic profiles do not predict differences in clinical response (Supplementary Fig. S18).

To identify the cell type responsible for the metabolic switch, we observed via immunofluorescence staining of representative tumors that the shift is predominantly occurring within PanCK+ cancer cells. This is evident by the high co-localization of glycolysis signals (PFK1) within PanCK+ cancer cells, with oxidative phosphorylation signals (ATP5E), however, being more broadly distributed across both PanCK+ cancer cells and noncancer cells (Supplementary Fig. S19). Whole-transcriptome DSP further supports this finding, whereby GSEA analysis only revealed the reciprocal expression of “oxidative phosphorylation” and “glycolysis” genes in the PanCK+ cancer regions but not in the immune T cells (CD3^+^ regions) or macrophage (CD68^+^ regions) compartments (Fig. 5D; Supplementary Table S7). Finally, metabolic pathway mapping in these PanCK+ cancer regions showed that whereas glycolytic reactions continued to remain elevated, there was, however, a notable change in the oxidative phosphorylation components. This is characterized by a shift to the complexes I and II and the electron-transfer flavoprotein oxidoreductase, with a downregulation of ATP synthase levels in the PanCK+ cells (Fig. 5E).

Lastly, as metabolic adaptation of tumors is a hallmark of a DTP state that enables their survival during treatment (35), we noted that neoadjuvant gefitinib tumors displayed elevated levels of DTP lung alveolar regeneration signatures, as well as signatures observed in TKI-treated NSCLC tumors with residual disease (Supplementary Fig. S20A; ref. 36). In addition, we observed modest upregulation of fatty acid oxidation and reactive oxygen species pathway signatures, consistent with cell line DTP-associated programs described by Oren and colleagues (Supplementary Fig. S20B; ref. 37). These DTP signatures, however, were not significantly correlated with patient clinical features, suggesting that the presence of these signatures is unlikely to affect patient response or clinical outcomes (Supplementary Fig. S21).

Overall, our results uncover a dynamic metabolic state within the TME, with diverse metabolic reactions serving as key potential drivers of the initial tumor response to neoadjuvant gefitinib. This observed shift also underscores the adaptability of tumor cells and suggests that additional metabolic interventions may help enhance the efficacy of EGFR TKIs in *EGFR*-mutated NSCLCs.

## Discussion

We observed that neoadjuvant gefitinib in resectable early-stage *EGFR*-mutated NSCLC was safe and feasible. Only one patient experienced a ≥G3 treatment-related AE, there were no treatment-related delays of planned surgery, and all patients had an uncomplicated surgical resection. The ORR was 62%, and pathologic downstaging was seen in 46% of patients. However, MPR was only seen in one (8%) patient, consistent with other recently reported studies of neoadjuvant osimertinib (9, 10). However, this is in contrast to studies evaluating neoadjuvant immune checkpoint inhibition in which relatively higher rates of MPR are associated with lower rates of RECIST response (38). Similarly, in the metastatic NSCLC setting, higher ORRs are commonly seen with targeted therapies compared with immunotherapies, without necessarily translating to longer absolute median survival times (39–42). This further illustrates that there exist key differences in the dynamics and mechanisms of anti-tumor response for different classes of therapy in NSCLC. Consequently, interpretation of clinical outcomes from studies evaluating different therapies—particularly trials of systemic therapy with curative intent—must be considered in this context.

Clinical outcomes in our study were generally consistent with other studies of neoadjuvant EGFR TKIs. A similar study of neoadjuvant gefitinib for 6 weeks in resectable stages II to IIIA *EGFR*-mutated NSCLC (*n* = 33) demonstrated an ORR of 55% and MPR of 24% with no ≥G3 treatment-related AEs and an R0 resection rate of 88% (43). Neoadjuvant erlotinib was also evaluated in two separate studies of patients with stages IIIA to N2 *EGFR*-mutated NSCLC with an ORR of 42% to 54% and MPR of 10% (MPR not reported in one study; refs. 44, 45). Notably, only 68% to 73% of patients in these studies successfully underwent radical surgery. More recent studies of neoadjuvant osimertinib have similarly shown limited MPR rates of 15% to 26% (9, 11, 46). Collectively, the observed response rates in these neoadjuvant EGFR TKI studies are lower than might be expected, at least radiologically compared with the metastatic setting and pathologically compared with immunotherapies. This may be in part due to the duration of EGFR TKI therapy and use of first-generation EGFR TKI in some studies. However, despite the use of EGFR TKIs in advanced disease for over a decade, the true underlying determinants of residual disease or drug-tolerant persistence to EGFR TKIs still remain to be elucidated. Therefore, we sought to investigate this further with deep multiregion genomic and transcriptomic interrogation of resected tissue.

**Figure 6.**
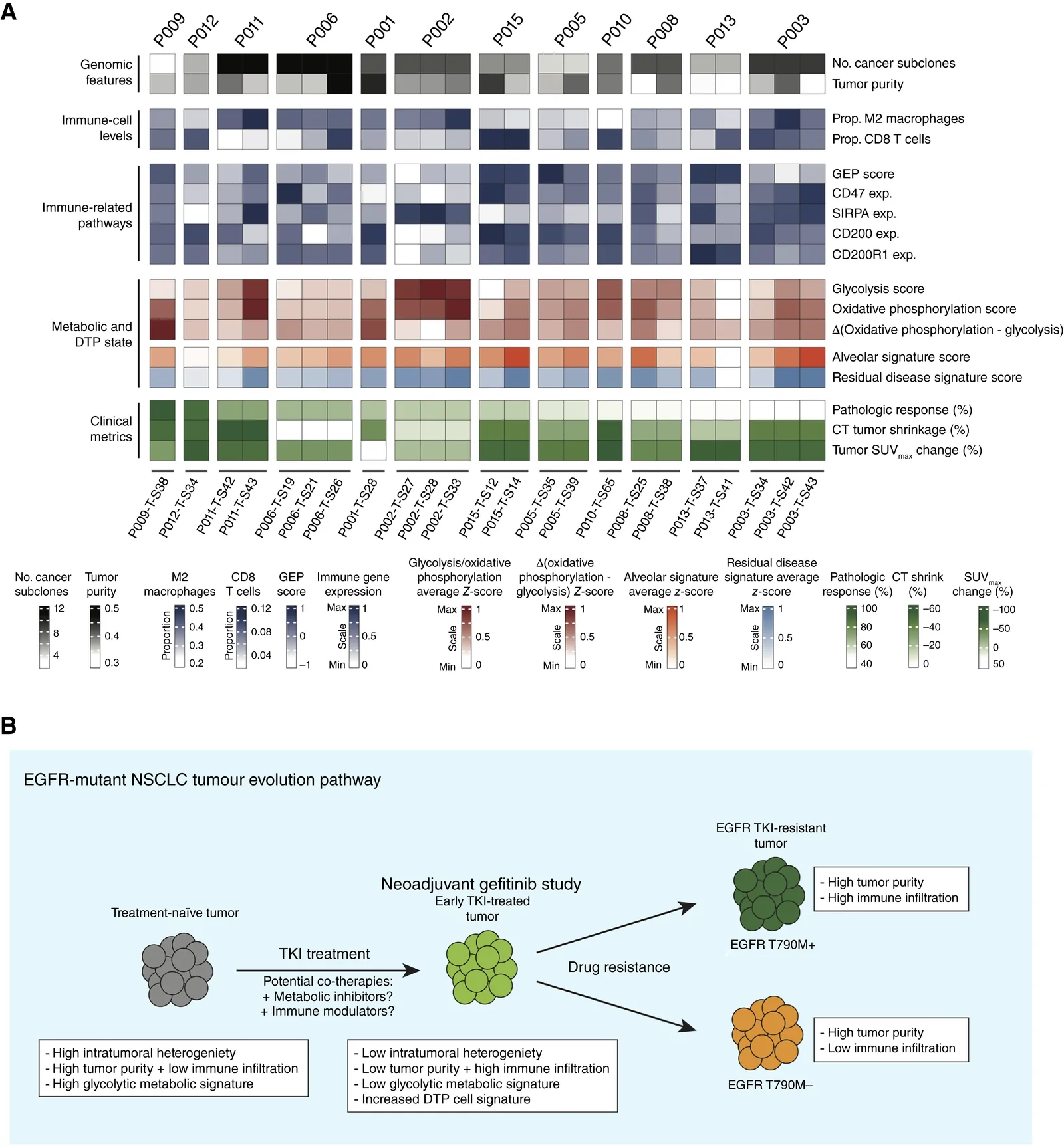
Comparative analysis and model for tumor evolution in neoadjuvant gefitinib-treated *EGFR*-mutated NSCLC tumors. **A,** Heatmap showing the qualitative differences between key findings (genomic, immune, metabolic, and DTP features) and clinical parameters in treatment-naïve and neoadjuvant gefitinib-treated tumors (**B**).

Our molecular analysis demonstrated that there were no genomic or transcriptomic features associated with various clinical measures of response, including CT, PET, and pathologic response (Fig. 6A). The major immune and metabolic features observed in the neoadjuvant gefitinib-treated tumors compared with the purity-matched treatment-naïve tumors are also illustrated in Fig. 6A. In addition, we found there was a distinct absence of known resistance mechanisms at 6 weeks of treatment, including the *EGFR* T790M mutation. Therefore, this analysis represents early adaptive changes, rather than mechanisms of acquired resistance. This provides supportive evidence for the presence of DTP cells which undergo further evolution over time and the acquisition of resistance mechanisms (47). The broad reduction in clonal diversity, mutational burden, and tumor purity across neoadjuvant therapy–treated tumors lends further weight to this paradigm—where this subpopulation of persister cells maintain tumor viability (48). Consistent with this, we also observed increased levels of DTP signatures in neoadjuvant gefitinib-treated tumors, further highlighting the potential role of this persister cell population in therapeutic resistance. Within this context, we identified smaller subclonal populations in neoadjuvant gefitinib tumors that may represent early precursors with the potential to expand into fully resistant clones after acquiring additional alterations. However, further functional validation in future studies may be crucial to confirm the presence of a true DTP phenotype, rather than reflecting more general stress responses (49). Eliminating this persister cell population early through combination therapeutic approaches therefore constitutes a crucial opportunity which may improve long-term outcomes (Fig. 6B). This may, in part, provide the underlying mechanistic rationale for improved survival outcomes with combination EGFR TKI and chemotherapy or amivantamab in the first-line setting for advanced *EGFR*-mutated NSCLC (3, 50, 51). To this end, our transcriptomic analysis further highlighted key features which may characterize the early adaptive changes in both the tumor and the local microenvironment and consequent therapeutic vulnerabilities.

The increased expression of immunoregulatory and inflammatory response genes hints at the potential rationale for adding immunomodulatory therapies, such as those targeting the CD47 or CD200 pathways (28, 29), or antiangiogenic agents (52). Although some of the tumor inflammation we observed may reflect a generic response to cell death, preclinical studies have shown that EGFR inhibition can modulate tumor–immune interactions, including PD-L1 induction, enhanced antigen presentation, and altered immune cell recruitment (52–54). Another study of neoadjuvant erlotinib also demonstrated remodeling of the TME after neoadjuvant therapy, with the potential implication of M2 polarization of tumor-associated macrophages (55). However, it should be noted that our understanding of macrophage biology is rapidly evolving, and their functional states may also be highly plastic (56). Chemotherapy is also known to induce broad immunologic changes in NSCLCs (57, 58), but as *EGFR*-mutated tumors are rarely treated with neoadjuvant chemotherapy, direct comparisons with EGFR TKI–treated tumors are limited. Within this context, our study does not aim to establish categorical differences between modalities but rather to describe the specific immune alterations following EGFR TKI therapy. In addition, the potential infiltration of CD8 T cells and M2-like macrophages should be interpreted as associations based on immune deconvolution, and evaluation with contemporary modalities such as single-cell sequencing in future studies would be helpful to establish true expansion of immune cell subsets. Much more comprehensive evaluation of the immune compartment may clarify the cell state of key immune cells and their functional impact on response, persistence, and resistance.

The observed metabolic shift from a glycolytic to oxidative phosphorylation elevated state may suggest that *EGFR*-mutated NSCLC may benefit from combinatorial drug-induced metabolic reprogramming or modulation during EGFR TKI therapy. However, conflicting results of trials with combination metformin plus EGFR TKI indicates the role of tumor metabolism in *EGFR*-mutated NSCLC remains incompletely understood (59–61). Although antibody–drug conjugates (ADC) hold therapeutic promise in advanced NSCLC, we did not observe minimal and inconsistent differences in the expression of selected ADC target genes between neoadjuvant gefitinib and treatment-naïve tumors (Supplementary Fig. S22), suggesting limited applicability in this context.

Finally, although our study provides evidence toward the efficacy and safety of neoadjuvant gefitinib, larger studies of neoadjuvant third-generation EGFR TKI therapy, and perhaps a longer duration of neoadjuvant therapy, are needed to confirm the clinical feasibility and efficacy of this neoadjuvant approach. Our study evaluated a first-generation EGFR TKI, and although differences with later-generation TKIs may be expected, the immune and metabolic adaptations we observed likely reflect a class effect of EGFR pathway inhibition rather than drug-specific phenomena. Thus, our findings remain relevant to osimertinib-based and future EGFR TKI strategies. Comparative analyses in emerging neoadjuvant osimertinib cohorts will be essential to validate these observations and extend their clinical applicability. Findings from studies of neoadjuvant osimertinib, with or without chemotherapy, will be crucial in this regard (9, 11, 48, 62). Further translational studies with deep sequencing will also be vital to completely recapitulate early tumor adaptations to EGFR TKI therapy. As we have demonstrated, however, analysis can be challenging, in particular due to changes in the local microenvironment leading to low tumor purities. Additional orthogonal validation of our findings, with newer technologies incorporating both spatial and single-cell sequencing capabilities, may be required.

### Conclusion

We have demonstrated that neoadjuvant gefitinib is safe and feasible in resectable *EGFR*-mutated NSCLC. However, limited pathologic response was observed. Deep genomic and transcriptomic sequencing, including multiregion and spatial analysis, provided unique insight into early adaptive response in both the tumor and local microenvironment. This uncovered the potential for rational combination approaches to improve therapeutic efficacy in *EGFR*-mutated NSCLC.

## Supplementary Material

Figure S1 Supplementary Figure 1. Survival outcomes of patients enrolled in the trial. (A) Disease-free survival (DFS) and (B) overall survival (OS).

Figure S2 Supplementary Figure 2. Genomic landscape of EGFR TKI resistance mutations in neoadjuvant gefitinib treated EGFR mutated tumours. (A) Oncoplot of EGFR TKI resistance genomic alterations in treatment-naive and neoadjuvant gefitinib cohorts. Significantly different gene mutation frequencies between the cohorts denoted by asterisks. (B) Cancer cell fraction estimates from EstimateClonality for EGFR T790M reads in treatment naive, neoadjuvant gefitinib and TKI-Resistant (51) cohorts (****: p<0.0001). (C) Comparison of tumour mutation burdens of treatment naive and neoadjuvant gefitinib cohorts at varying sequencing depths. Dashed line denotes samples with both 100x and 500x sequencing.

Figure S3 Supplementary Figure 3. Analysis of HER2 (ERBB2) amplifications in neoadjuvant gefitinib treated EGFR mutated tumours. (A) HER2 (ERBB2) gene amplification levels denoted as the difference between a sample specific GISTIC2 amplification threshold and the observed gene amplitude. Amplitude differences above 0 are classified as significantly altered levels. (B) Expression levels denoted in log counts per million (logCPM) of HER2 (ERBB2) amplified and copy number neutral samples in each cohort.

Figure S4 Supplementary Figure 4. Genomic landscape of 95 non-EGFR TKI resistance mutations found in neoadjuvant gefitinib treated EGFR mutated tumours. Significantly different mutation frequencies is denoted by asterisks (*: p<0.0001).

Figure S5 Supplementary Figure 5. Associations between mutations detected in neoadjuvant gefitinib treated EGFR mutated tumours and treatment response. (A) CT change from baseline (%), (B) SUVmax change from baseline (%), and (C) pathological response (%) stratified by mutation status in the genes ERBB2, LRBA, MDM2, PIK3CA, PTPN23 or TP53. P-values are from Wilcoxon rank sum tests.

Figure S6 Supplementary Figure 6. Measures of clonal diversity in treatment naive and neoadjuvant gefitinib patients. (A-B) Two estimates for clone diversity in purity-matched cohorts, which consider phylogenetic structure (Clonal & Ancestor Diversity Index) and the mutational burden (Clonal & Mutational Diversity Index) for each patient. (C) Number of subclones detected for each patient tumour at matched depth and matched purity.

Figure S7 Supplementary Figure 7. Inferred clone phylogenies with tumour purity for treatment naive patients. Phylogenetic trees of detected clones from Treatment Naive patients, as inferred by CITUP. Only known LUAD (n=96) and Cancer Gene Census (n=359) driver genes are annotated for each clone on the tree. The mutation burden of a given clone is denoted by the vertical distance between itself and its parent node, whereas the horizontal distances between the nodes do not correspond to genetic distances.

Figure S8 Supplementary Figure 8. Inferred clone phylogenies with pathological response (%) and tumour purity for neoadjuvant gefitinib patients. Phylogenetic trees of detected clones from treatment naive patients, as inferred by CITUP. Only known LUAD (n=96) and Cancer Gene Census (n=359) driver genes are annotated for each clone on the tree. The mutation burden of a given clone is denoted by the vertical distance between itself and its parent node, whereas the horizontal distances between the nodes do not correspond to genetic distances.

Figure S9 Supplementary Figure 9. EGFR gene amplification and expression levels. (A) Percentages of EGFR amplifications for each of the treatment naive, neoadjuvant gefitinib and TKI-Resistant (51) cohorts as estimated by GISTIC2. P-values denote Chi-squared tests comparing the number of high amplification samples as a proportion of its cohort, against another cohort. (B) The number of EGFR mutant alleles estimated by EstimateClonality for EGFR amplified, and neutral samples. (C) Expression levels denoted in log counts per million (logCPM) of EGFR amplified and copy number neutral samples in each cohort.

Figure S10 Supplementary Figure 10. Correlation plots of the tumour genomic features TMB, tumour purity, and number of tumour subclones against neoadjuvant patient clinical parameters. (A) CT tumour shrinkage (%), (B) Tumour SUVmaxChange (%), (C) Pathological response (%). The Pearson correlation coefficient between each two compared features is as presented with its corresponding p-value within the plot. Each point represents the median molecular feature value across tumour sectors from the same patient.

Figure S11 Supplementary Figure 11. Additional boxplots of CIBERSORTx celltype proportions comparing all tumour samples present within the treatment naïve vs. neoadjuvant gefitinib cohort.

Figure S12 Supplementary Figure 12. Heatmap of purity-matched immune cell types estimated by CIBERSORTx ranked by the order and direction of significance (adjusted p-value) between treatment naïve vs. neoadjuvant gefitinib cohorts.

Figure S13 Supplementary Figure 13. Additional boxplots of CIBERSORTx celltype proportions comparing only purity-matched tumour samples present within the treatment naïve vs. neoadjuvant gefitinib cohort.

Figure S14 Supplementary Figure 14. Cytokine/chemokine and immune checkpoint responses in purity-matched neoadjuvant gefitinib NSCLC tumours. (A) Gene expression boxplots of the NK and T-cell ligands PVR and PVRL2 (CD112), as well as the NK and T-cell receptors TIGIT, CD226, CD96 and PVRIG (CD112R). (B) Gene expression boxplot of the OX40L-OX40 immune checkpoint ligand-receptor pair. (C) Boxplot of gene expression levels comparing CCL5-CCR5 and CXCL12-CXCR4 ligand receptor pairs in treatment naïve vs neoadjuvant gefitinib tumours.

Figure S15 Supplementary Figure 15. Correlation analysis of CD47-SIRPA and CD200-CD200R1 checkpoint expression (LogCPM) against neoadjuvant gefitinib patient clinical parameters (A) Correlation of CD47-SIRPA expression with CT change from baseline (%), SUVmax change from baseline (%), and pathological response (%). (B) Correlation of CD200-CD200R1 expression with CT change from baseline (%), SUVmax change from baseline (%), and pathological response (%).

Figure S16 Supplementary Figure 16. Correlation analysis of M2 Macrophage and CD8 T-cell tumour proportions (from CIBERSORTx) against neoadjuvant gefitinib patient clinical parameters. (A) Correlation of M2 Macrophage proportion with CT change from baseline (%), SUVmax change from baseline (%), and pathological response (%). (B) Correlation of tumour CD8 T-cell proportion with CT change from baseline (%), SUVmax change from baseline (%), and pathological response (%).

Figure S17 Supplementary Figure 17. Expression changes of CD47-SIRPA and CD200-CD200R1 from pre-treatment (Treatment naive), on-treatment (neoadjuvant gefitinib) and upon EGFR TKI-resistance. (A) LogCPM values of CD47-SIRPA and CD200-CD200R1 in all samples. (B) LogCPM values of CD47-SIRPA and CD200-CD200R1 in the purity-matched cohort.

Figure S18 Supplementary Figure 18. Correlation analysis metabolic gene expression signatures with neoadjuvant gefitinib patient clinical parameters. (A) Correlation of Glycolysis signature score vs. CT change from baseline (%), SUVmax change from baseline (%), and pathological response (%). (B) Correlation of Oxidative Phosphorylation score vs. CT change from baseline (%), SUVmax change from baseline (%), and pathological response (%).

Figure S19 Supplementary Figure 19. Analysis of glycolytic and oxidative phosphorylation signatures in neoadjuvant gefitinib or treatment naive tumours. (A) Immunofluorescence (IF) co-staining of treatment naive or neoadjuvant gefitinib tumour sections with α-PFK1 (Glycolysis), α-ATP5E (Oxidative Phosphorylation), α-PanCK (Cancer Cells) and DAPI (cell nuclei). (B-C) Co-localization analysis of metabolic IF staining intensities in treatment naive vs. neoadjuvant gefitinib tumours.

Figure S20 Supplementary Figure 20. Expression of drug-tolerant persister (DTP) gene signatures in treatment naïve and neoadjuvant gefitinib tumours. (A) Boxplots of DTP lung alveolar cell and EGFR TKI residual disease signature scores in purity-matched cohorts, as defined by Maynard et al. (66). (B) Boxplots of fatty acid oxidation (FAO) and reactive oxygen species (ROS) pathway signature scores in purity-matched cohorts, as defined by Oren et al. (67).

Figure S21 Supplementary Figure 21. Correlation of DTP gene signature scores with clinical response parameters in neoadjuvant gefitinib treated tumours. Correlations of (A) DTP lung alveolar cell signatures, (B) EGFR TKI residual disease signatures, (C) ROS scores and (D) FAO scores with CT change from baseline (%), SUVmax change from baseline (%), and pathological response (%).

Figure S22 Supplementary Figure 22. Expression of clinically actionable ADC genes (A) LogCPM values of ADC genes using all samples. (B) LogCPM values of ADC genes in the purity-matched cohort.

Figure S23 Supplementary Figure 23. Tumour and nodal downstaging with neoadjuvant gefitinib; Sankey diagram depicting the tumour and nodal status changes from pre-neoadjuvant gefitinib (left) to post-neoadjuvant gefitinib (right)

Figure S24 Supplementary Figure 24. Comparison of T-cell functional phenotype score in tumour, border and stromal regions from whole trascriptome digital spatial profiling

Figure S25 Supplementary Figure 25. Evaluation of M1 and M2 macrophages in tumour, border and stromal regions from whole trascriptome digital spatial profiling comparing neoadjuvant gefitinib treated versus treatment naïve tumours

Table S1 Supplementary Table 1. Treatment-related adverse events

Table S2 Supplementary Table 2. Pre- and post-treatment primary tumour size and staging

Table S3 Supplementary Table 3. List of Treatment Naive and Neoadjuvant Gefitinib DNA and RNA samples (All samples and Purity-matched cohorts)

Table S4 Supplementary Table 4. SMuRF variant calls in Treatment Naïve and Neoadjuvant Gefitinib tumours

Table S5 Supplementary Table 5. GISTIC2 output of LUAD driver genes in Treatment Naive and Neoadjuvant Gefitinib tumours

Table S6 Supplementary Table 6. Normalized LogCPM counts of expressed genes in Treatment Naive and Neoadjuvant Gefitinib tumours (All-samples and Purity-matched)

Table S7 Supplementary Table 7. List of differentially expressed genes between Treatment Naive and Neoadjuvant Gefitinib tumours (PanCK+, CD3 and CD68 regions)

Table S8 Supplementary Table 8. Table of Mutation Cellular Prevalences in the Treatment Naive patient cohort

Table S9 Supplementary Table 9. Table of Mutation Cellular Prevalences in the Neoadjuvant Gefitinib patient cohort

Table S10 Supplementary Table 10. Representativeness of Study Participants

Supplementary Methods Supplementary Methods

## Data Availability

Raw sequencing data for neoadjuvant gefitinib and treatment-naïve (63) *EGFR*-mutated NSCLCs have been deposited in the European Genome-phenome Archive (accession numbers EGAC00001003182 and EGAS00001006942, respectively). The NanoString GeoMx spatial transcriptomic and other data generated during this study are available from the corresponding author upon reasonable request, subject to institutional policies and applicable data-sharing agreements.
