# Supplementary material for: Early EGFR Inhibition Triggers Actionable Tumor and Microenvironmental Remodeling in Lung Cancer
Source: Cancer Res Commun. 2026 Aug 26;6(8):1993–2009. doi: 10.1158/2767-9764.CRC-26-0073 (PMC13510642; doi:10.1158/2767-9764.CRC-26-0073)
Supplement: Supplementary file 1 [file crc-26-0073_figure_s10_suppsf10.pdf]

Supp. Figure 10

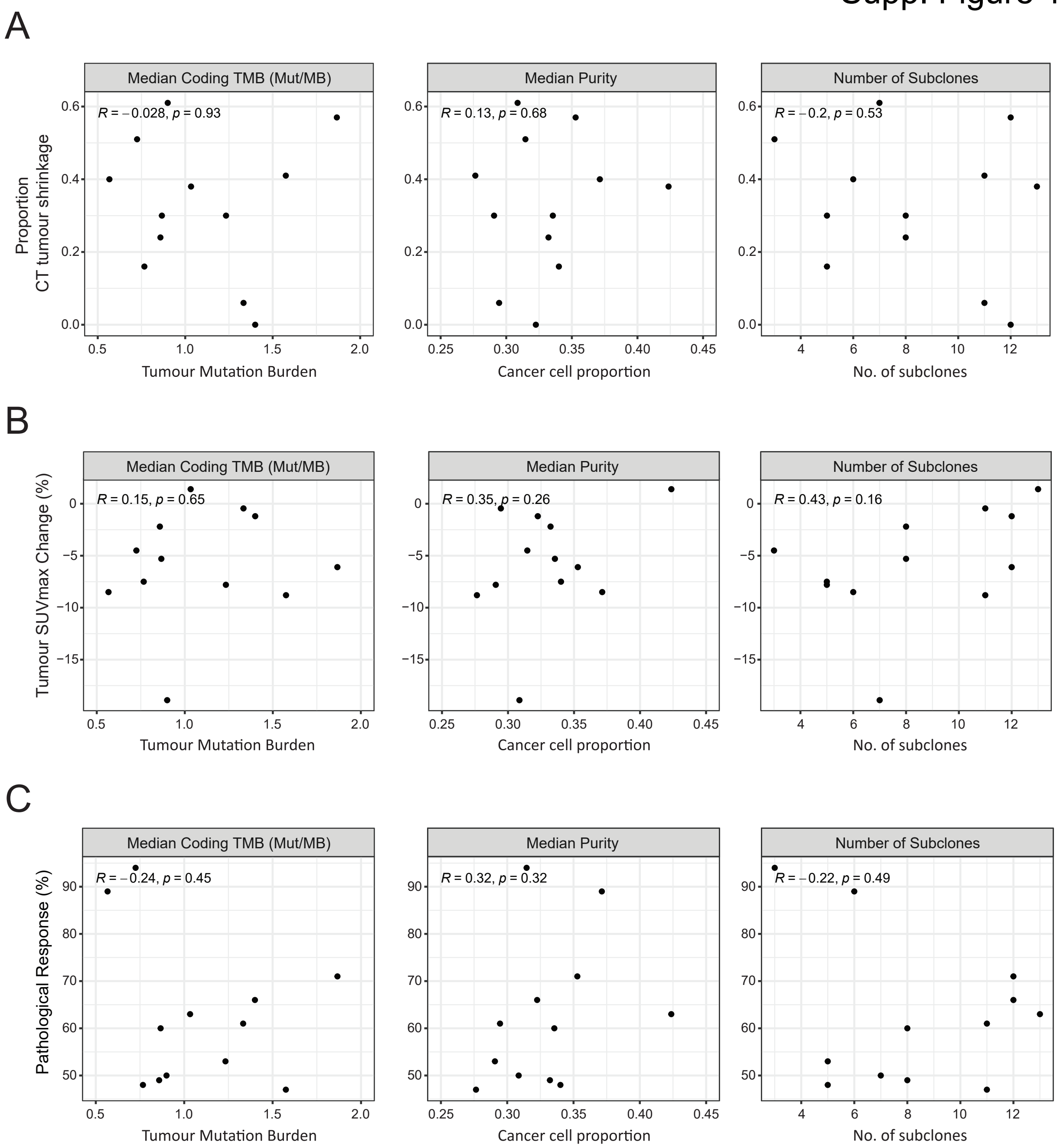

Supplementary Figure 10. Correlation plots of the tumour genomic features TMB, tumour purity, and number of tumour subclones against neoadjuvant patient clinical parameters. (A) CT tumour shrinkage (%), (B) Tumour SUVmaxChange (%), (C) Pathological response (%). The Pearson correlation coefficient between each two compared features is as presented with its corresponding p-value within the plot. Each point represents the median molecular feature value across tumour sectors from the same patient.
