# Supplementary material for: Early EGFR Inhibition Triggers Actionable Tumor and Microenvironmental Remodeling in Lung Cancer
Source: Cancer Res Commun. 2026 Aug 26;6(8):1993–2009. doi: 10.1158/2767-9764.CRC-26-0073 (PMC13510642; doi:10.1158/2767-9764.CRC-26-0073)
Supplement: Supplementary file 3 [file crc-26-0073_figure_s12_suppsf12.pdf]

Supp. Figure 12

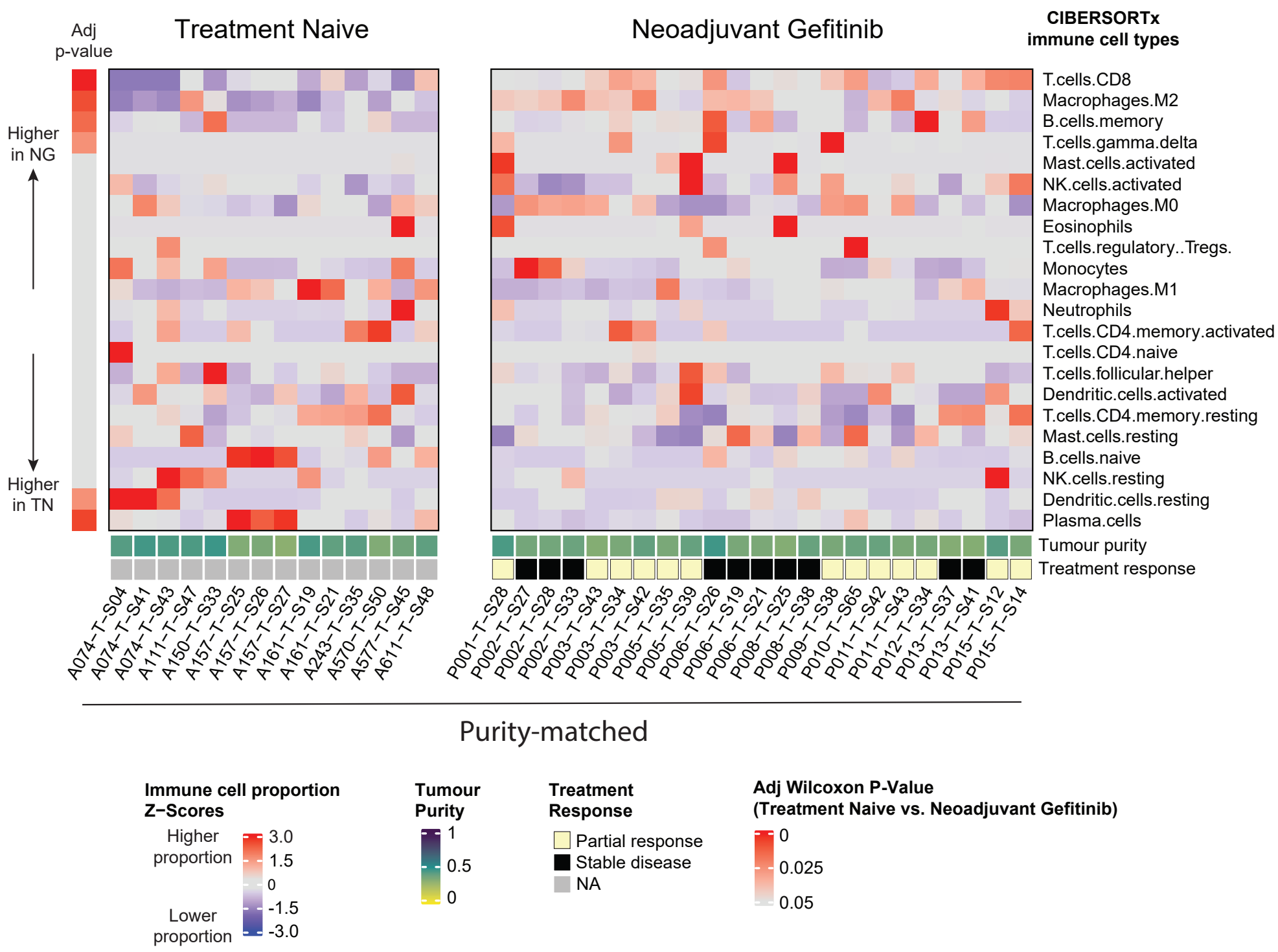

Supplementary Figure 12. Heatmap of purity-matched immune cell types estimated by CIBERSORTx ranked by the order and direction of significance (adjusted p-value) between treatment naïve vs. neoadjuvant gefitinib cohorts.
