# Supplementary material for: Early EGFR Inhibition Triggers Actionable Tumor and Microenvironmental Remodeling in Lung Cancer
Source: Cancer Res Commun. 2026 Aug 26;6(8):1993–2009. doi: 10.1158/2767-9764.CRC-26-0073 (PMC13510642; doi:10.1158/2767-9764.CRC-26-0073)
Supplement: Supplementary file 4 [file crc-26-0073_figure_s13_suppsf13.pdf]

Supp. Figure 13

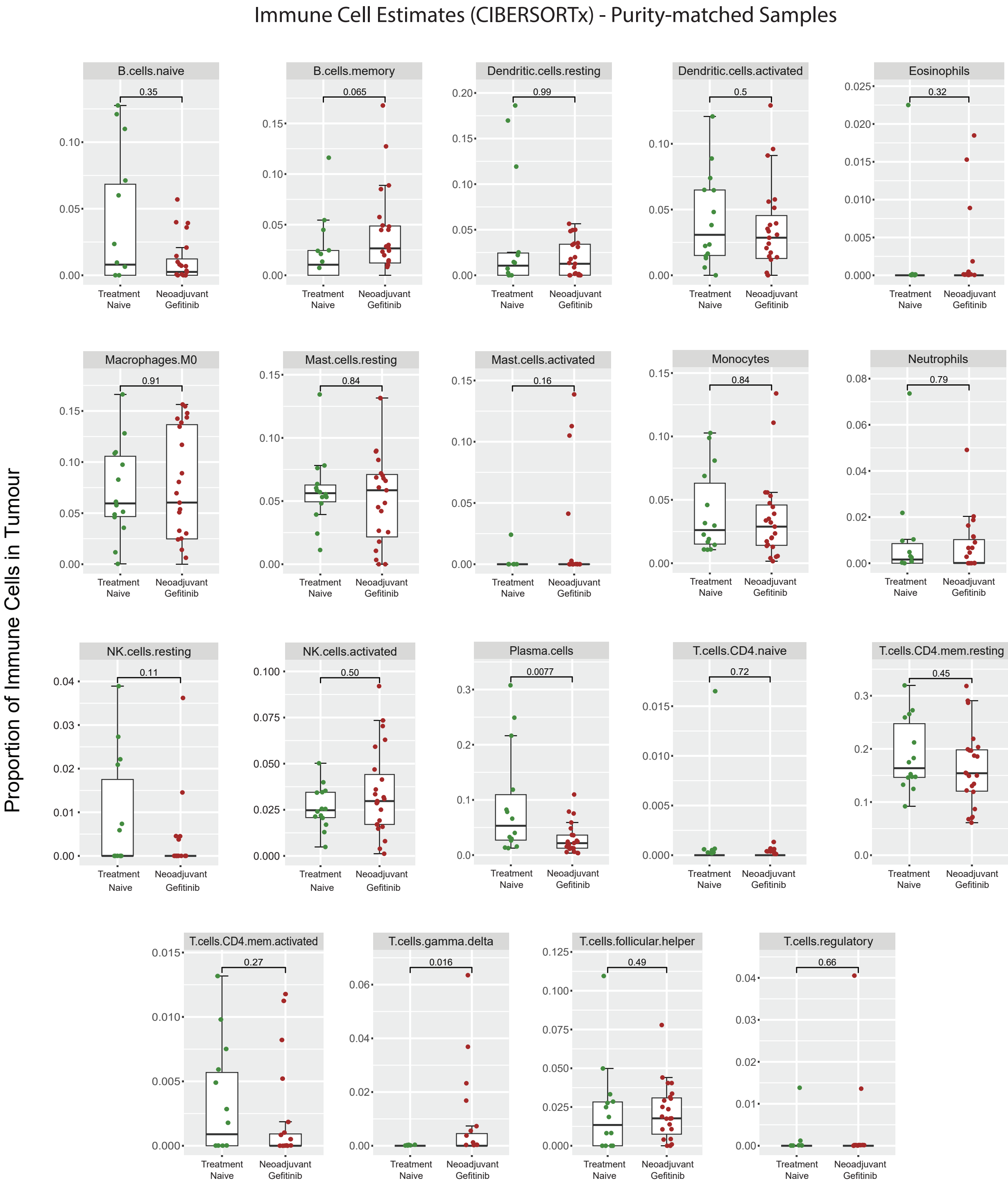

Supplementary Figure 13. Additional boxplots of CIBERSORTx celltype proportions comparing only purity-matched tumour samples present within the treatment naïve vs. neoadjuvant gefitinib cohort.
