# Supplementary material for: Early EGFR Inhibition Triggers Actionable Tumor and Microenvironmental Remodeling in Lung Cancer
Source: Cancer Res Commun. 2026 Aug 26;6(8):1993–2009. doi: 10.1158/2767-9764.CRC-26-0073 (PMC13510642; doi:10.1158/2767-9764.CRC-26-0073)
Supplement: Supplementary file 5 [file crc-26-0073_figure_s14_suppsf14.pdf]

Supp. Figure 14

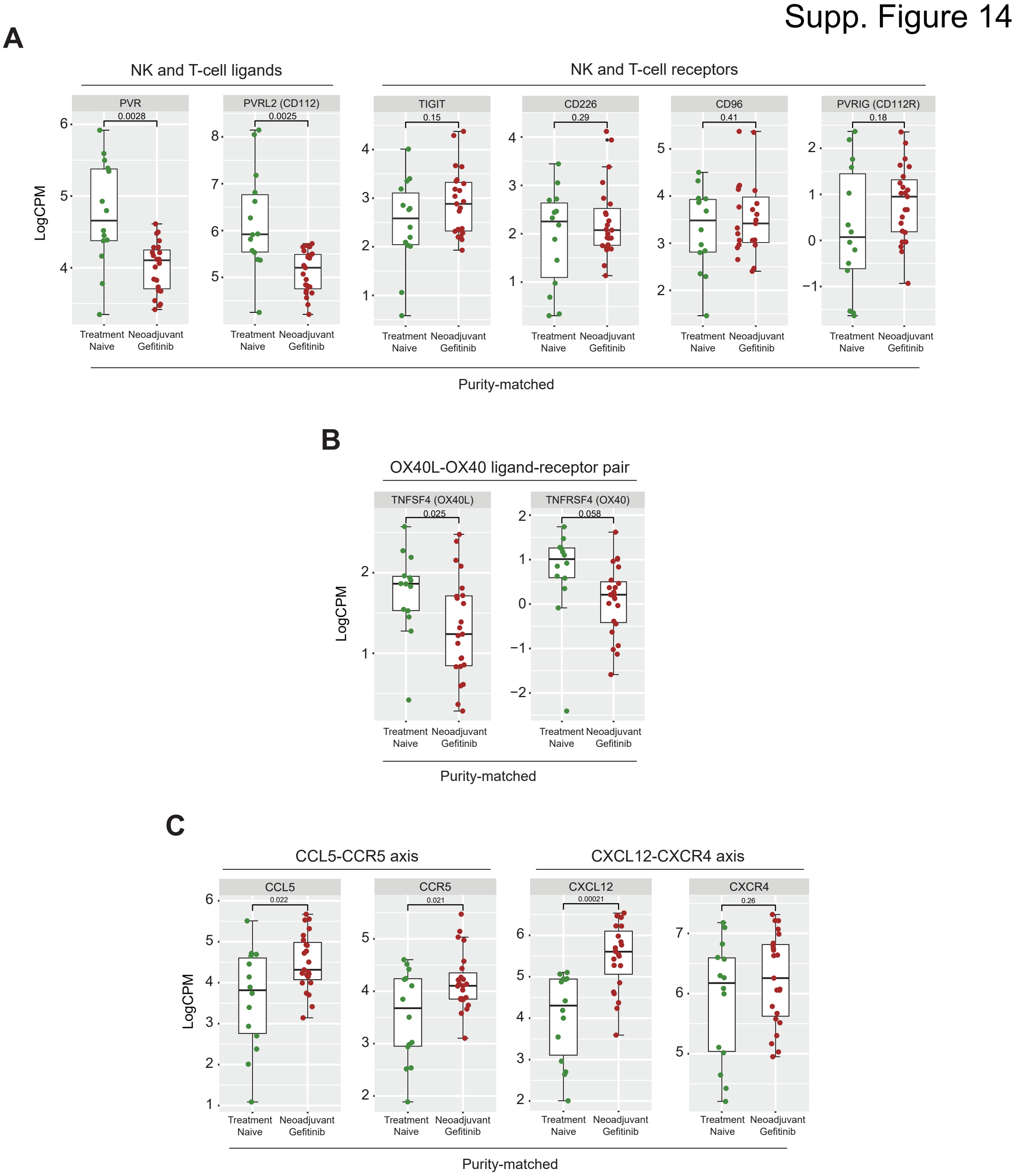

Supplementary Figure 14. Cytokine/chemokine and immune checkpoint responses in purity-matched neoadjuvant gefitinib NSCLC tumours. (A) Gene expression boxplots of the NK and T-cell ligands PVR and PVRL2 (CD112), as well as the NK and T-cell receptors TIGIT, CD226, CD96 and PVRIG (CD112R). (B) Gene expression boxplot of the OX40L-OX40 immune checkpoint ligand-receptor pair. (C) Boxplot of gene expression levels comparing CCL5-CCR5 and CXCL12-CXCR4 ligand receptor pairs in treatment naïve vs neoadjuvant gefitinib tumours.
