# Supplementary material for: Early EGFR Inhibition Triggers Actionable Tumor and Microenvironmental Remodeling in Lung Cancer
Source: Cancer Res Commun. 2026 Aug 26;6(8):1993–2009. doi: 10.1158/2767-9764.CRC-26-0073 (PMC13510642; doi:10.1158/2767-9764.CRC-26-0073)
Supplement: Supplementary file 6 [file crc-26-0073_figure_s15_suppsf15.pdf]

Supp. Figure 15

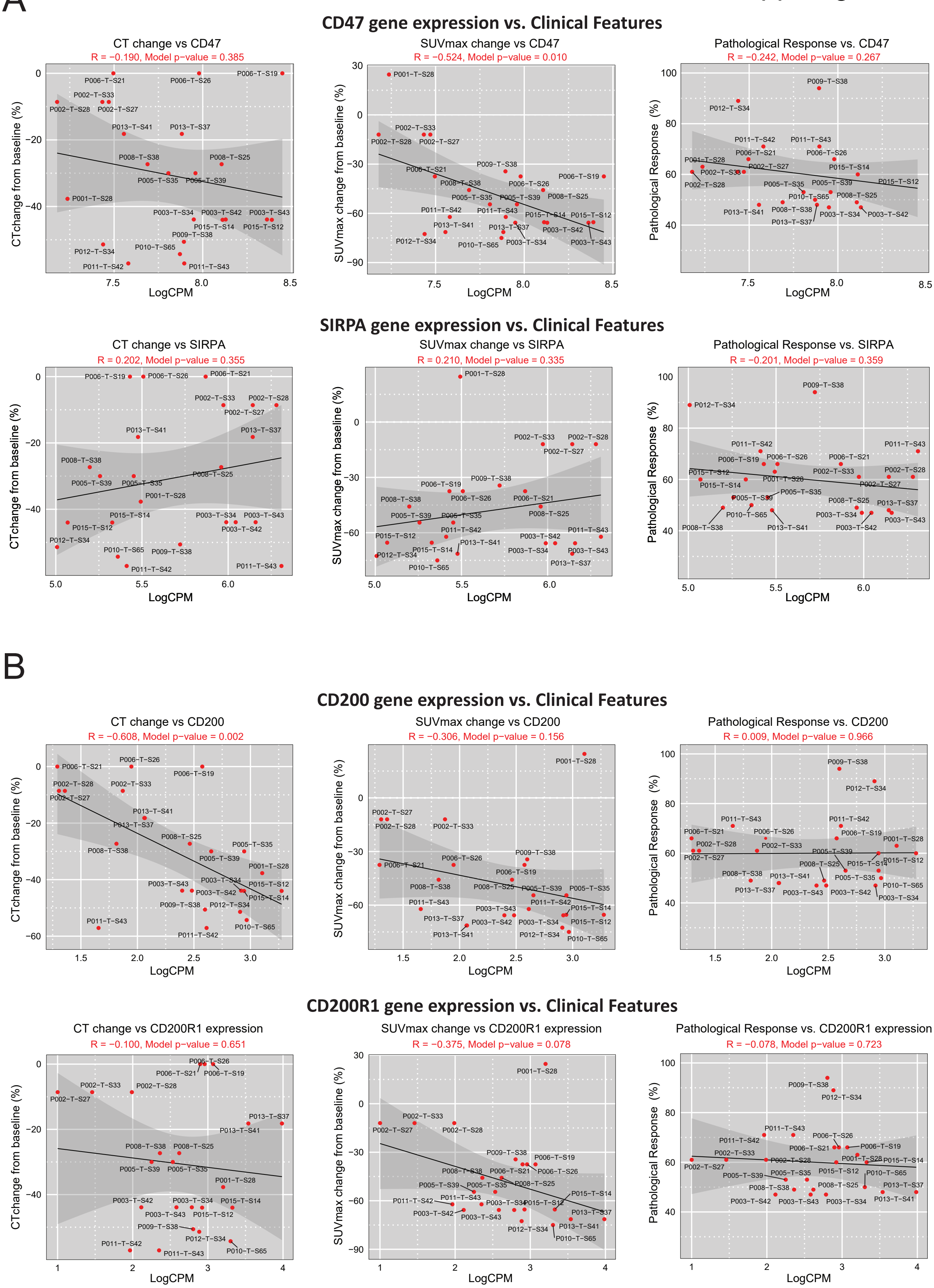

Supplementary Figure 15. Correlation analysis of CD47-SIRPA and CD200-CD200R1 checkpoint expression (LogCPM) against neoadjuvant gefitinib patient clinical parameters (A) Correlation of CD47-SIRPA expression with CT change from baseline (%), SUVmax change from baseline (%), and pathological response (%). (B) Correlation of CD200-CD200R1 expression with CT change from baseline (%), SUVmax change from baseline (%), and pathological response (%).
