# Supplementary material for: Early EGFR Inhibition Triggers Actionable Tumor and Microenvironmental Remodeling in Lung Cancer
Source: Cancer Res Commun. 2026 Aug 26;6(8):1993–2009. doi: 10.1158/2767-9764.CRC-26-0073 (PMC13510642; doi:10.1158/2767-9764.CRC-26-0073)
Supplement: Supplementary file 7 [file crc-26-0073_figure_s16_suppsf16.pdf]

Supp. Figure 16

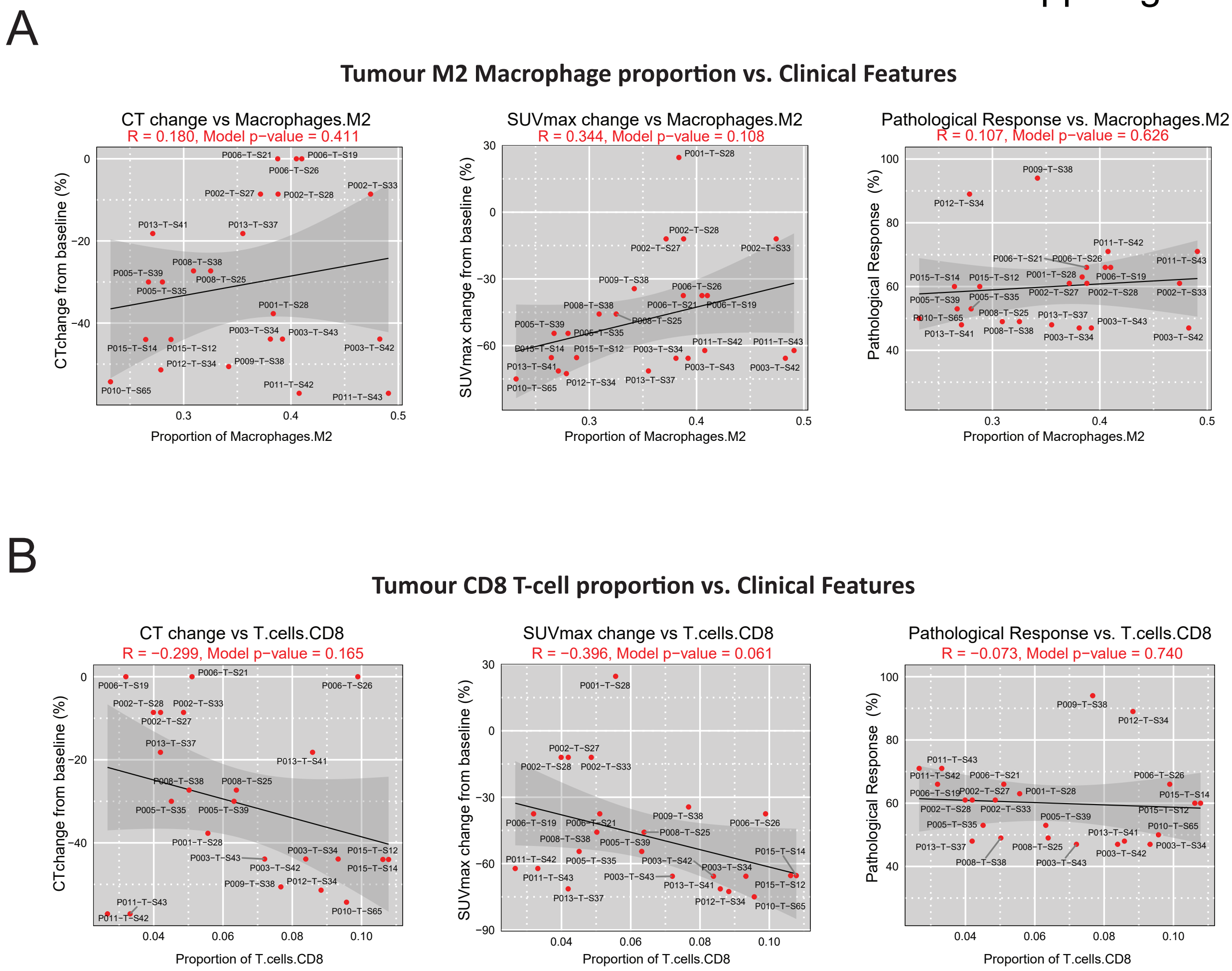

Supplementary Figure 16. Correlation analysis of M2 Macrophage and CD8 T-cell tumour proportions (from CIBERSORTx) against neoadjuvant gefitinib patient clinical parameters. (A) Correlation of M2 Macrophage proportion with CT change from baseline (%), SUVmax change from baseline (%), and pathological response (%). (B) Correlation of tumour CD8 T-cell proportion with CT change from baseline (%), SUVmax change from baseline (%), and pathological response (%).
