# Supplementary material for: Early EGFR Inhibition Triggers Actionable Tumor and Microenvironmental Remodeling in Lung Cancer
Source: Cancer Res Commun. 2026 Aug 26;6(8):1993–2009. doi: 10.1158/2767-9764.CRC-26-0073 (PMC13510642; doi:10.1158/2767-9764.CRC-26-0073)
Supplement: Supplementary file 8 [file crc-26-0073_figure_s17_suppsf17.pdf]

Supp. Figure 17

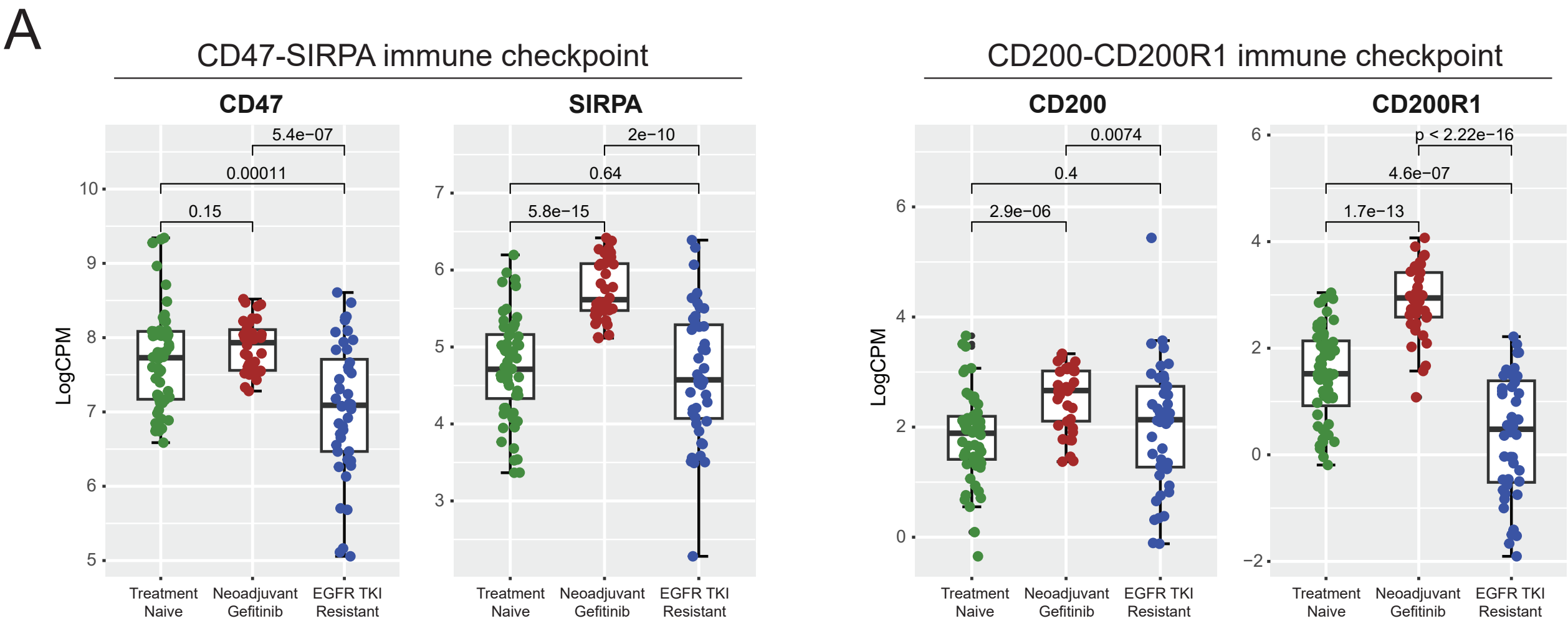

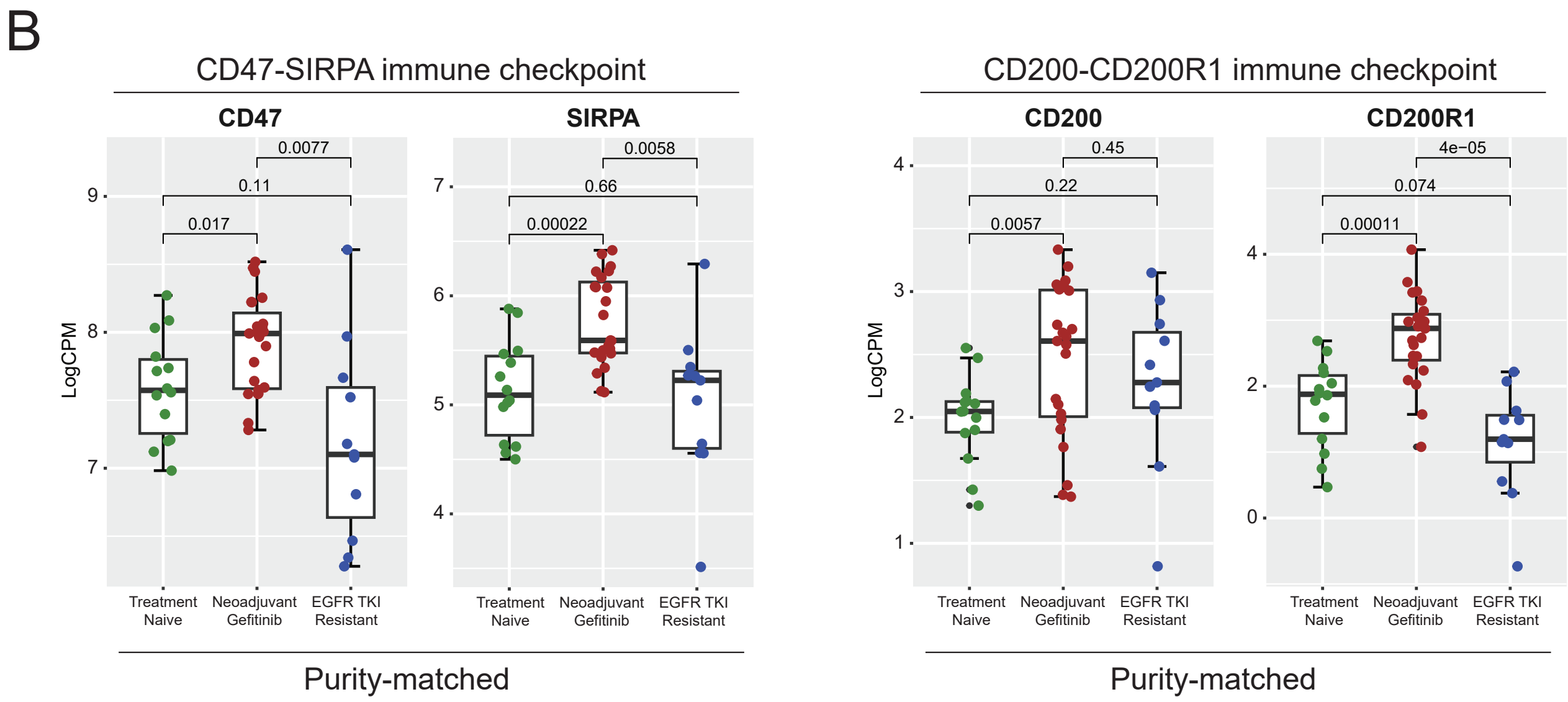

Supplementary Figure 17. Expression changes of CD47-SIRPA and CD200-CD200R1 from pre-treatment (Treatment naive), on-treatment (neoadjuvant gefitinib) and upon EGFR TKI-resistance. (A) LogCPM values of CD47-SIRPA and CD200-CD200R1 in all samples. (B) LogCPM values of CD47-SIRPA and CD200-CD200R1 in the purity-matched cohort.
