# Supplementary material for: Early EGFR Inhibition Triggers Actionable Tumor and Microenvironmental Remodeling in Lung Cancer
Source: Cancer Res Commun. 2026 Aug 26;6(8):1993–2009. doi: 10.1158/2767-9764.CRC-26-0073 (PMC13510642; doi:10.1158/2767-9764.CRC-26-0073)
Supplement: Supplementary file 10 [file crc-26-0073_figure_s19_suppsf19.pdf]

Supp. Figure 19

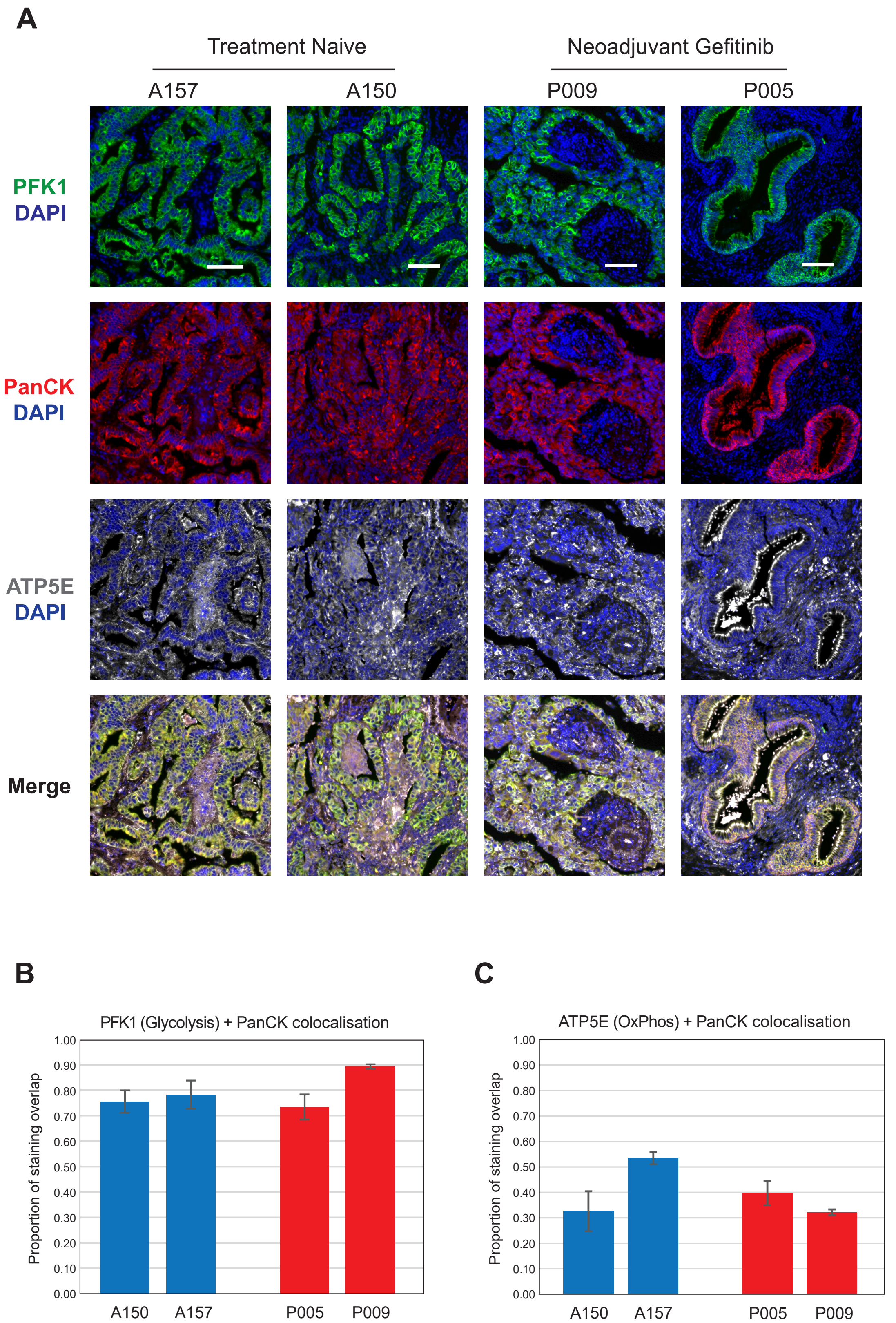

Supplementary Figure 19. Analysis of glycolytic and oxidative phosphorylation signatures in neoadjuvant gefitinib or treatment naive tumours. (A) Immunofluorescence (IF) co-staining of treatment naive or neoadjuvant gefitinib tumour sections with α-PFK1 (Glycolysis), α-ATP5E (Oxidative Phosphorylation), α-PanCK (Cancer Cells) and DAPI (cell nuclei). (B-C) Co-localization analysis of metabolic IF staining intensities in treatment naive vs. neoadjuvant gefitinib tumours.
