# Supplementary material for: Early EGFR Inhibition Triggers Actionable Tumor and Microenvironmental Remodeling in Lung Cancer
Source: Cancer Res Commun. 2026 Aug 26;6(8):1993–2009. doi: 10.1158/2767-9764.CRC-26-0073 (PMC13510642; doi:10.1158/2767-9764.CRC-26-0073)
Supplement: Supplementary file 11 [file crc-26-0073_figure_s1_suppsf1.pdf]

Supp. Figure 1

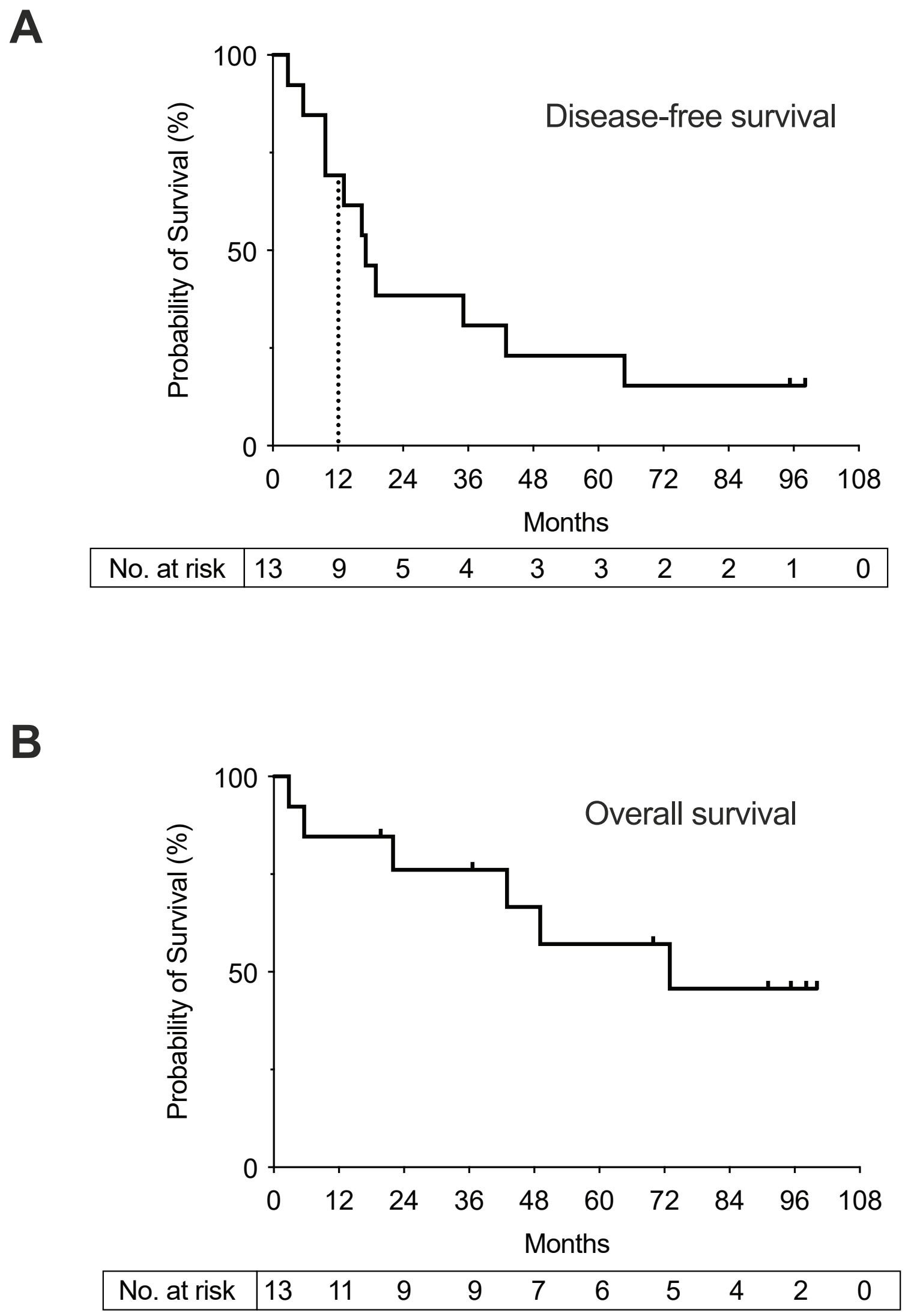

Supplementary Figure 1. Survival outcomes of patients enrolled in the trial. (A) Disease-free survival (DFS) and (B) overall survival (OS).
