# Supplementary material for: Early EGFR Inhibition Triggers Actionable Tumor and Microenvironmental Remodeling in Lung Cancer
Source: Cancer Res Commun. 2026 Aug 26;6(8):1993–2009. doi: 10.1158/2767-9764.CRC-26-0073 (PMC13510642; doi:10.1158/2767-9764.CRC-26-0073)
Supplement: Supplementary file 12 [file crc-26-0073_figure_s20_suppsf20.pdf]

Supp. Figure 20

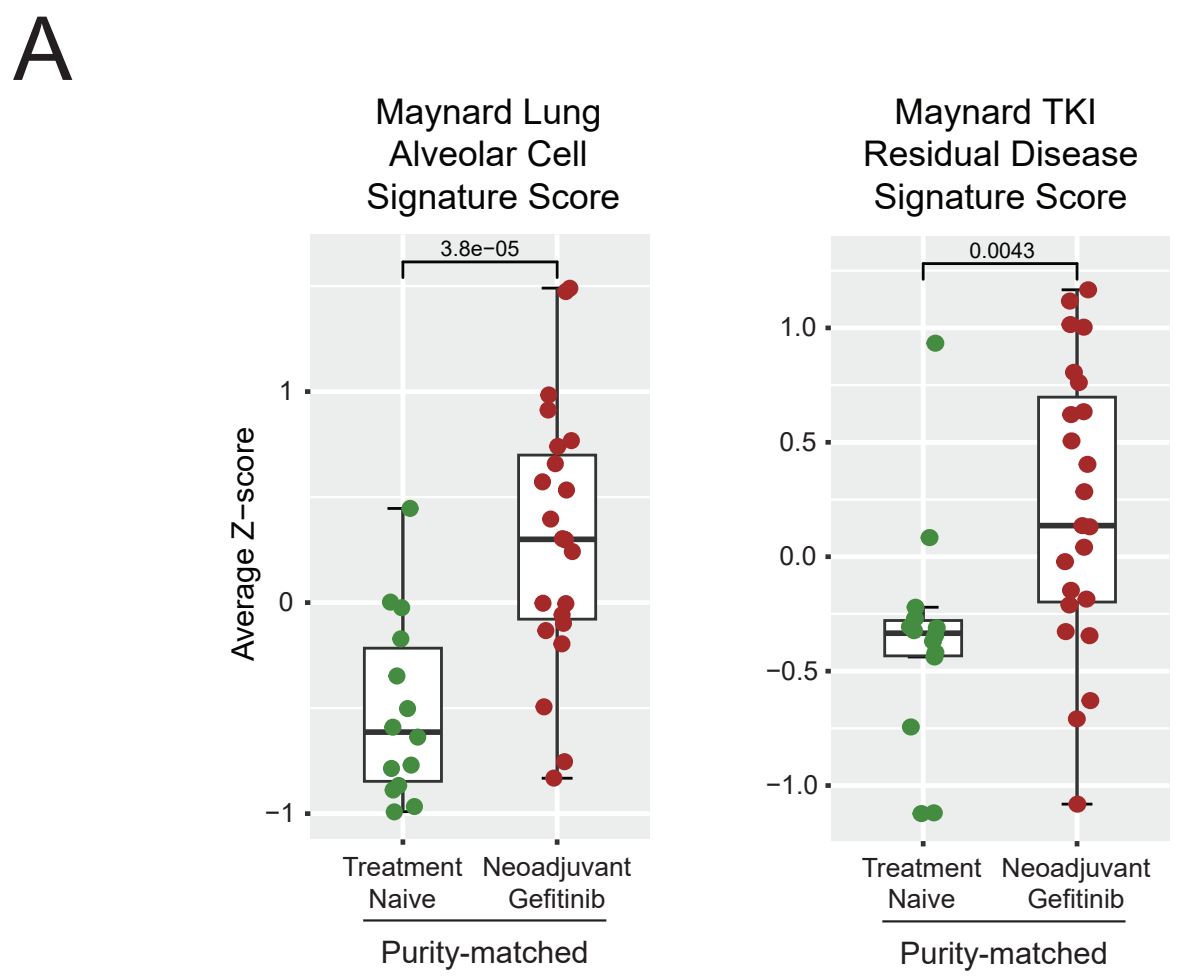

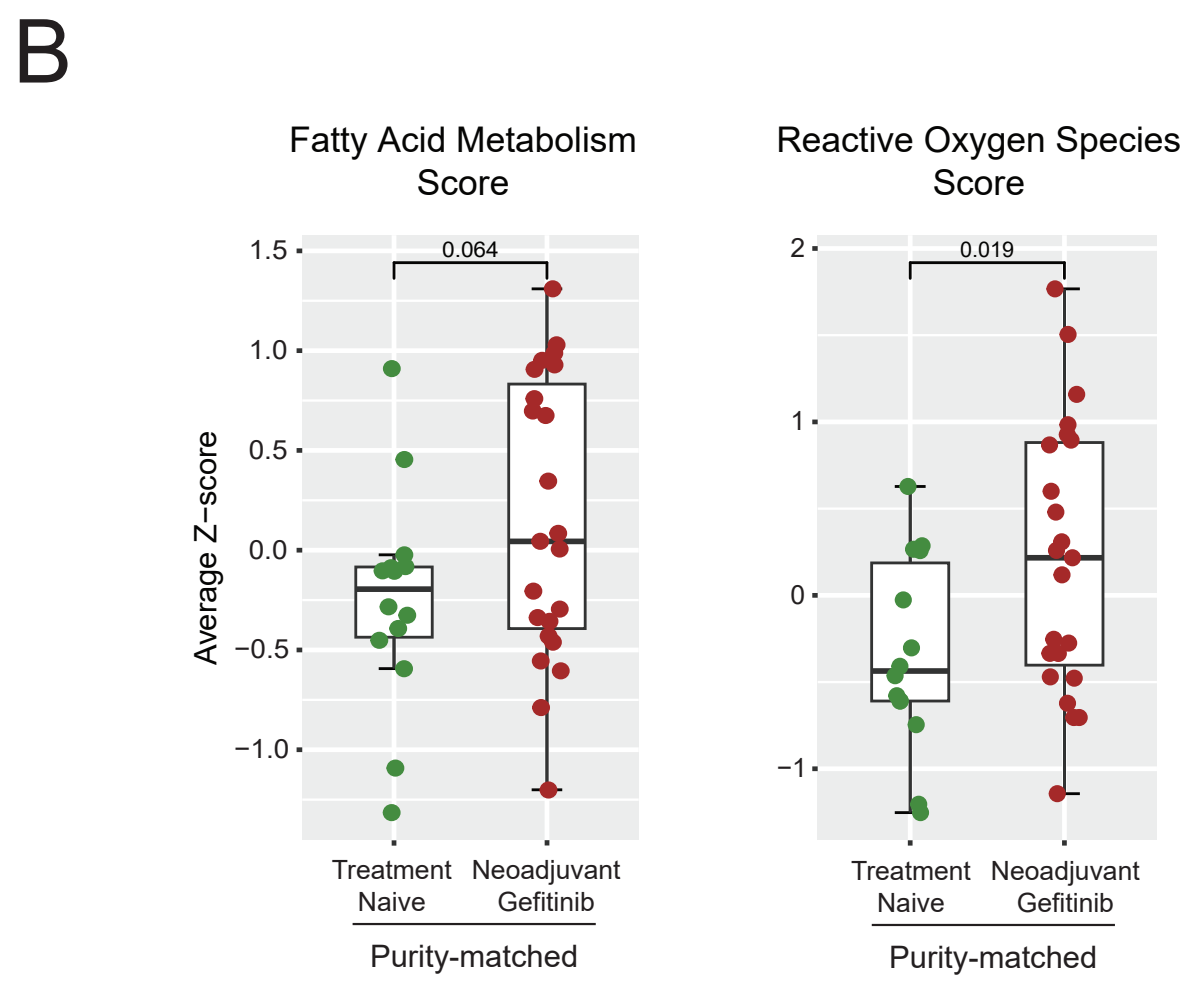

Supplementary Figure 20. Expression of drug-tolerant persister (DTP) gene signatures in treatment naïve and neoadjuvant gefitinib tumours. (A) Boxplots of DTP lung alveolar cell and EGFR TKI residual disease signature scores in purity-matched cohorts, as defined by Maynard et al. . (B) Boxplots of fatty acid oxidation (FAO) and reactive oxygen species (ROS) pathway signature scores in purity-matched cohorts, as defined by Oren et al. .
