# Supplementary material for: Early EGFR Inhibition Triggers Actionable Tumor and Microenvironmental Remodeling in Lung Cancer
Source: Cancer Res Commun. 2026 Aug 26;6(8):1993–2009. doi: 10.1158/2767-9764.CRC-26-0073 (PMC13510642; doi:10.1158/2767-9764.CRC-26-0073)
Supplement: Supplementary file 13 [file crc-26-0073_figure_s21_suppsf21.pdf]

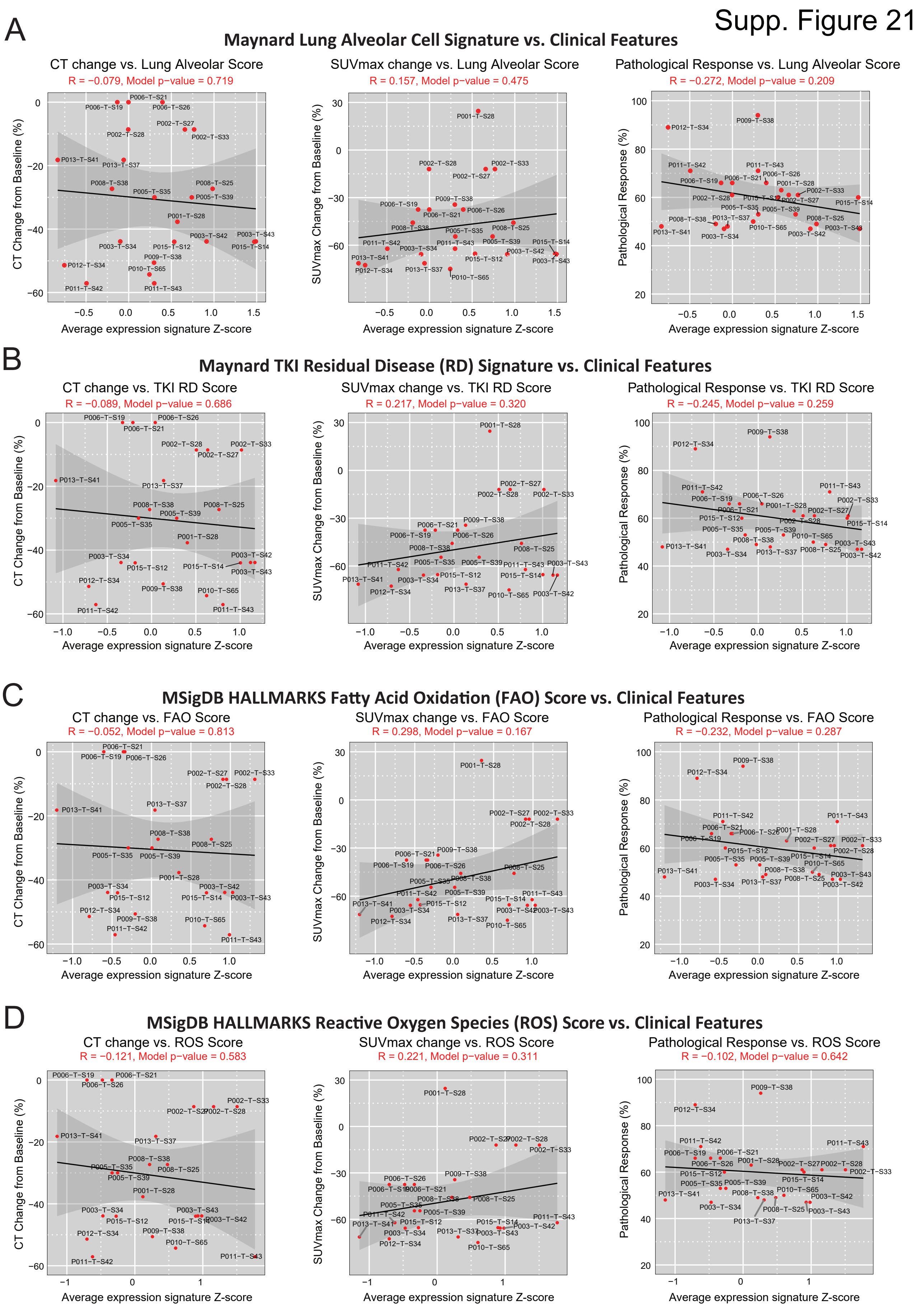

Supplementary Figure 21. Correlation of DTP gene signature scores with clinical response parameters in neoadjuvant gefitinib treated tumours. Correlations of (A) DTP lung alveolar cell signatures, (B) EGFR TKI residual disease signatures, (C) ROS scores and (D) FAO scores with CT change from baseline (%), SUVmax change from baseline (%), and pathological response (%)..
