# Supplementary material for: Early EGFR Inhibition Triggers Actionable Tumor and Microenvironmental Remodeling in Lung Cancer
Source: Cancer Res Commun. 2026 Aug 26;6(8):1993–2009. doi: 10.1158/2767-9764.CRC-26-0073 (PMC13510642; doi:10.1158/2767-9764.CRC-26-0073)
Supplement: Supplementary file 14 [file crc-26-0073_figure_s22_suppsf22.pdf]

Supp. Figure 22

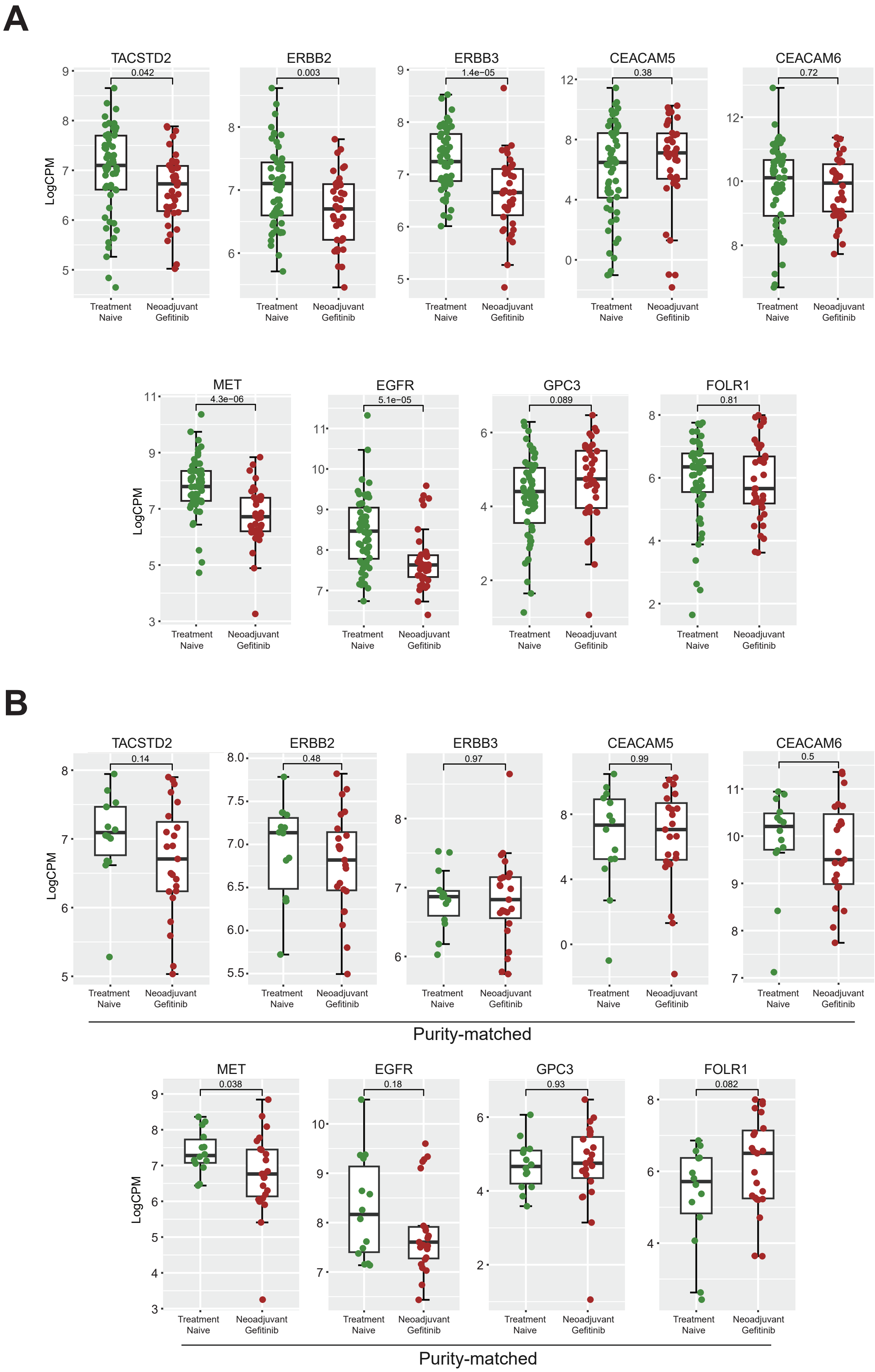

Supplementary Figure 22. Expression of clinically actionable ADC genes (A) LogCPM values of ADC genes using all samples. (B) LogCPM values of ADC genes in the purity-matched cohort.
