# Supplementary material for: Early EGFR Inhibition Triggers Actionable Tumor and Microenvironmental Remodeling in Lung Cancer
Source: Cancer Res Commun. 2026 Aug 26;6(8):1993–2009. doi: 10.1158/2767-9764.CRC-26-0073 (PMC13510642; doi:10.1158/2767-9764.CRC-26-0073)
Supplement: Supplementary file 15 [file crc-26-0073_figure_s23_suppsf23.pdf]

Supp. Figure 23

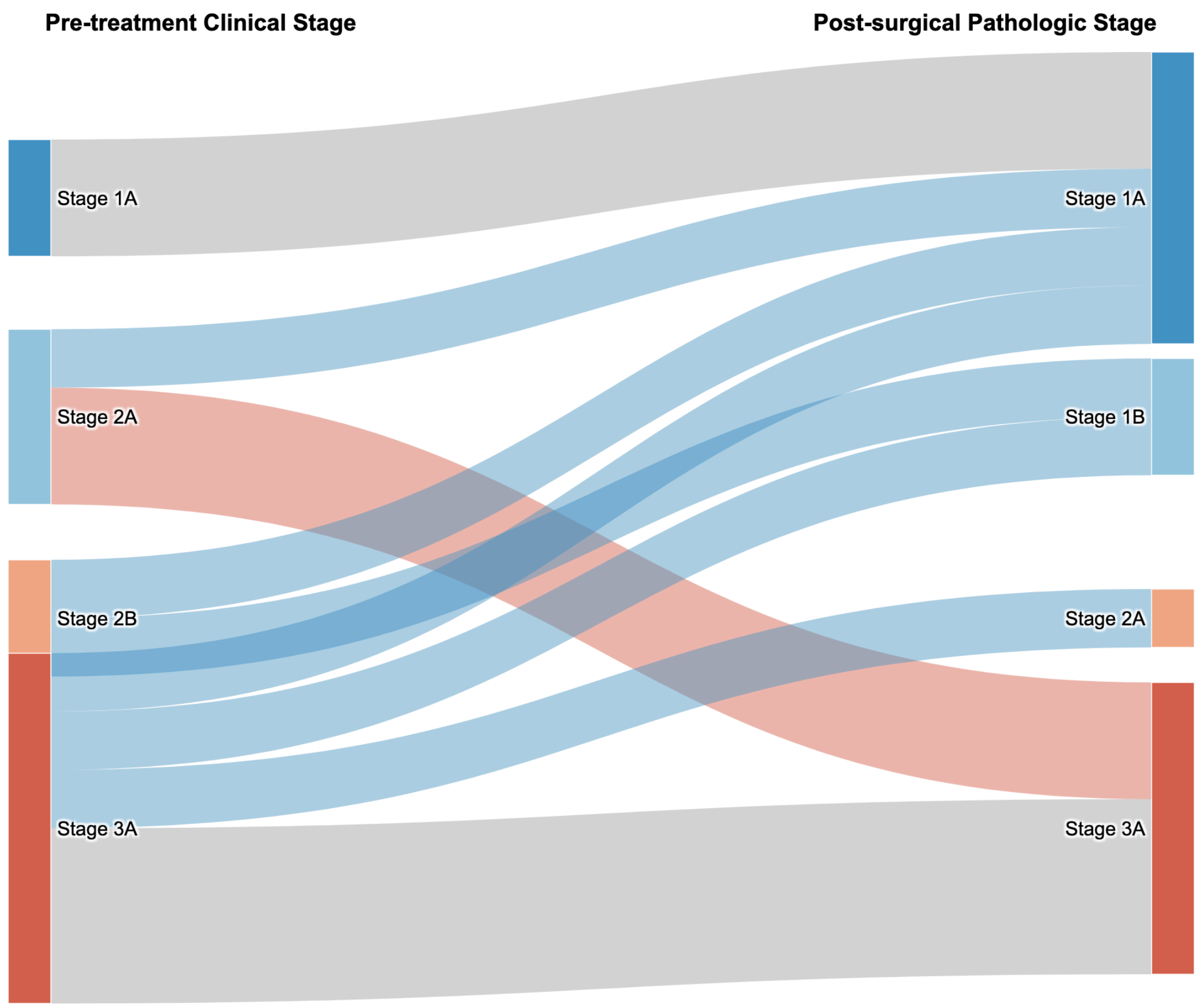

Supplementary Figure 23. Tumour and nodal downstaging with neoadjuvant gefitinib; Sankey diagram depicting the tumour and nodal status changes from pre-neoadjuvant gefitinib (left) to post-neoadjuvant gefitinib (right)
