# Supplementary material for: Early EGFR Inhibition Triggers Actionable Tumor and Microenvironmental Remodeling in Lung Cancer
Source: Cancer Res Commun. 2026 Aug 26;6(8):1993–2009. doi: 10.1158/2767-9764.CRC-26-0073 (PMC13510642; doi:10.1158/2767-9764.CRC-26-0073)
Supplement: Supplementary file 16 [file crc-26-0073_figure_s24_suppsf24.pdf]

Supp. Figure 24

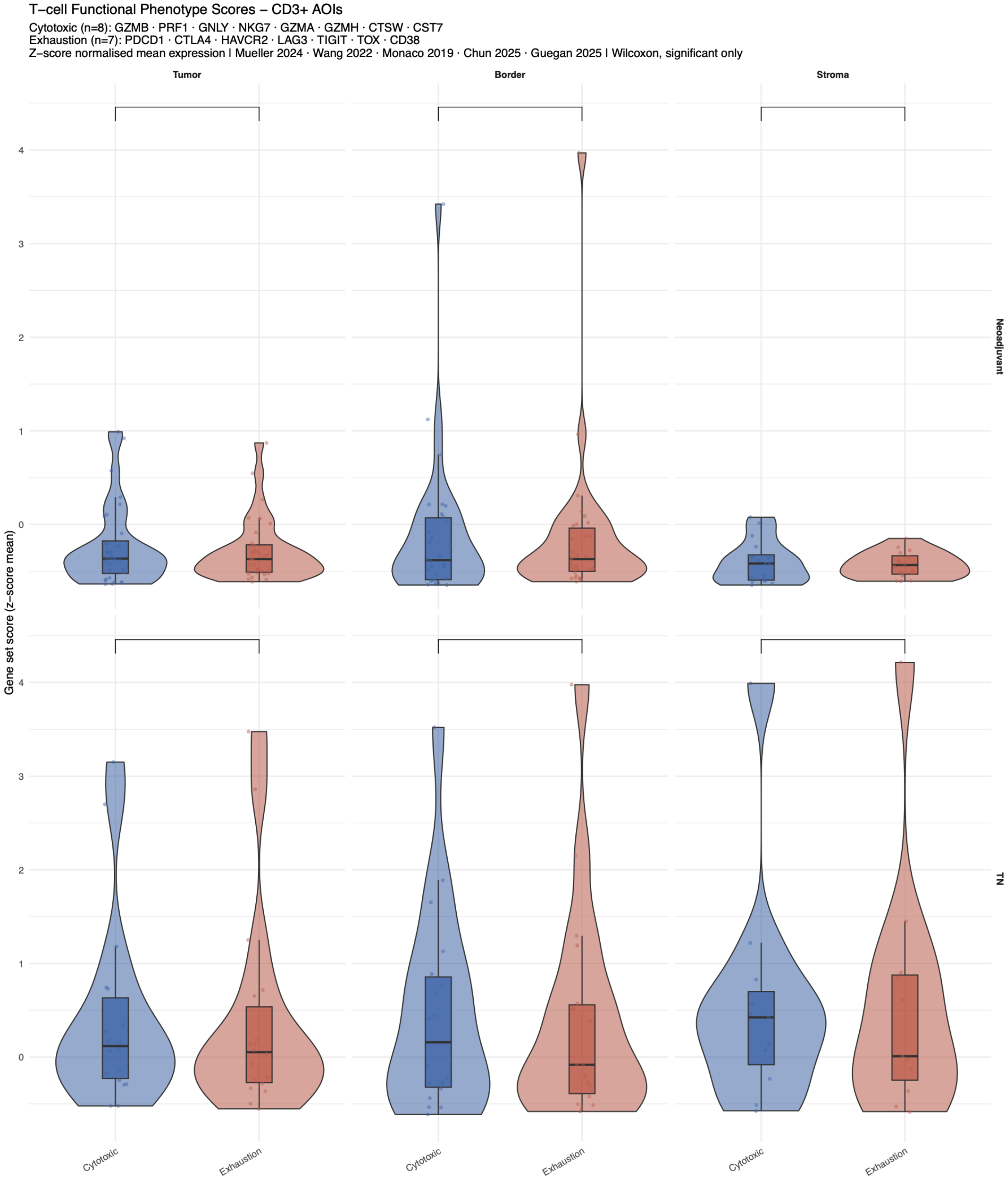

Supplementary Figure 24. Comparison of T-cell functional phenotype score in tumour, border and stromal regions from whole trascriptome digital spatial profiling
