# Supplementary material for: Early EGFR Inhibition Triggers Actionable Tumor and Microenvironmental Remodeling in Lung Cancer
Source: Cancer Res Commun. 2026 Aug 26;6(8):1993–2009. doi: 10.1158/2767-9764.CRC-26-0073 (PMC13510642; doi:10.1158/2767-9764.CRC-26-0073)
Supplement: Supplementary file 17 [file crc-26-0073_figure_s25_suppsf25.pdf]

Supp. Figure 25

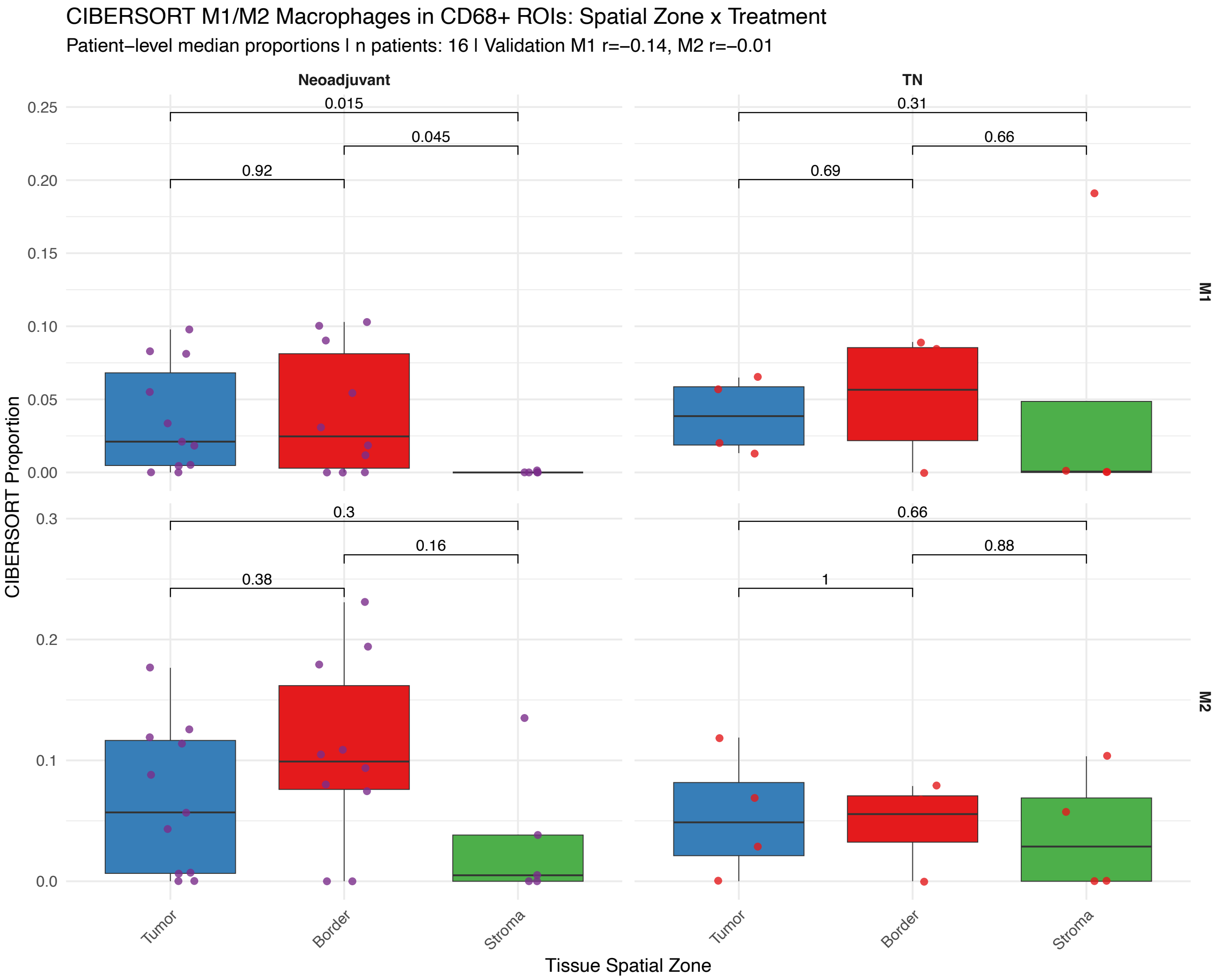

Supplementary Figure 25. Evaluation of M1 and M2 macrophages in tumour, border and stromal regions from whole trascriptome digital spatial profiling comparing neoadjuvant gefitinib treated versus treatment naïve tumours
