# Supplementary material for: Early EGFR Inhibition Triggers Actionable Tumor and Microenvironmental Remodeling in Lung Cancer
Source: Cancer Res Commun. 2026 Aug 26;6(8):1993–2009. doi: 10.1158/2767-9764.CRC-26-0073 (PMC13510642; doi:10.1158/2767-9764.CRC-26-0073)
Supplement: Supplementary file 18 [file crc-26-0073_figure_s2_suppsf2.pdf]

Supp. Figure 2

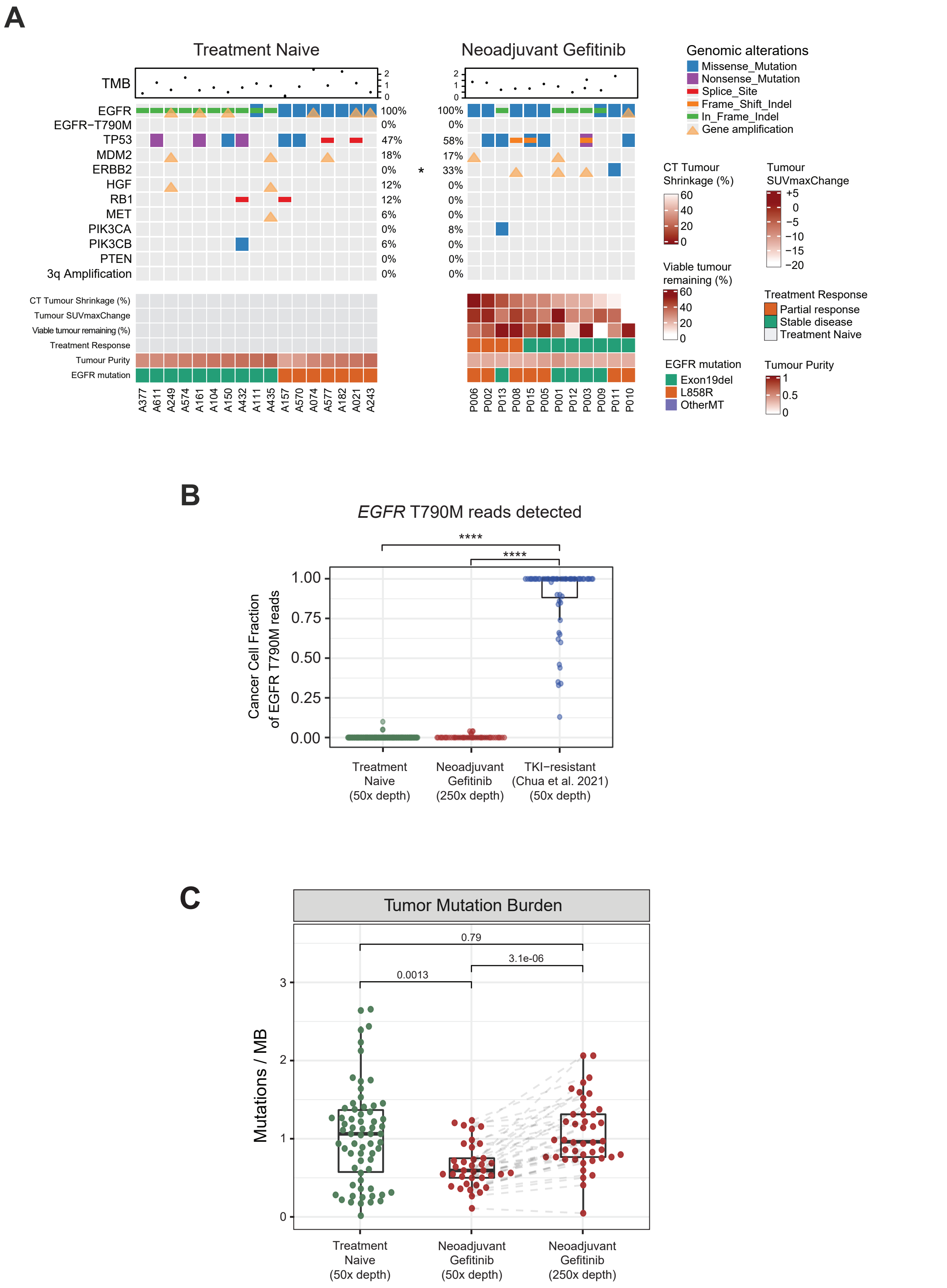

Supplementary Figure 2. Genomic landscape of EGFR TKI resistance mutations in neoadjuvant gefitinib treated *EGFR* mutated tumours. (A) Oncoplot of EGFR TKI resistance genomic alterations in treatment-naive and neoadjuvant gefitinib cohorts. Significantly different gene mutation frequencies between the cohorts denoted by asterisks. (B) Cancer cell fraction estimates from EstimateClonality for EGFR T790M reads in treatment naive, neoadjuvant gefitinib and TKI-Resistant cohorts (****: p<0.0001). (C) Comparison of tumour mutation burdens of treatment naive and neoadjuvant gefitinib cohorts at varying sequencing depths. Dashed line denotes samples with both 100x and 500x sequencing.
