# Supplementary material for: Early EGFR Inhibition Triggers Actionable Tumor and Microenvironmental Remodeling in Lung Cancer
Source: Cancer Res Commun. 2026 Aug 26;6(8):1993–2009. doi: 10.1158/2767-9764.CRC-26-0073 (PMC13510642; doi:10.1158/2767-9764.CRC-26-0073)
Supplement: Supplementary file 19 [file crc-26-0073_figure_s3_suppsf3.pdf]

Supp. Figure 3

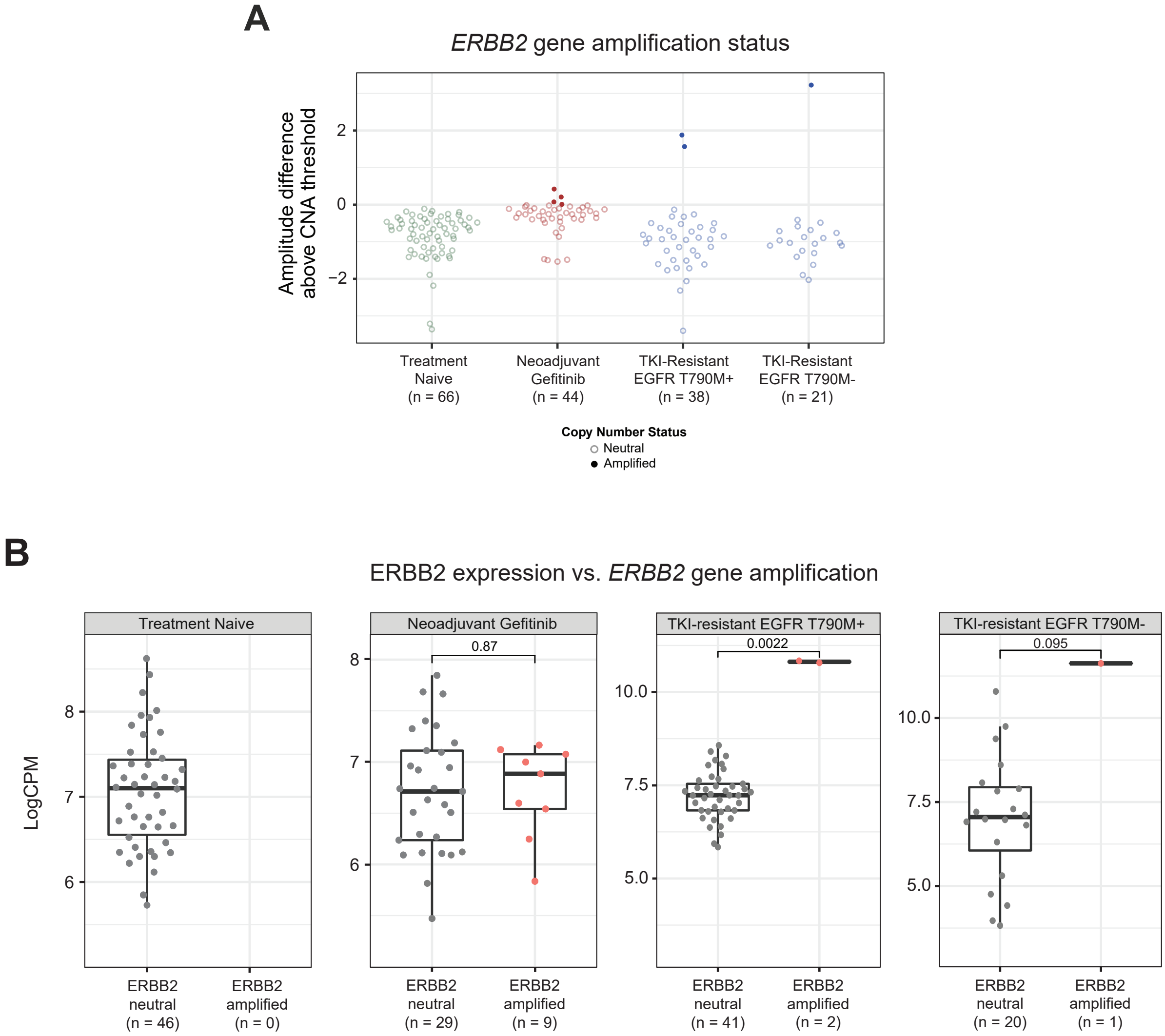

Supplementary Figure 3. Analysis of HER2 (ERBB2) amplifications in neoadjuvant gefitinib treated *EGFR* mutated tumours. (A) HER2 (ERBB2) gene amplification levels denoted as the difference between a sample specific GISTIC2 amplification threshold and the observed gene amplitude. Amplitude differences above 0 are classified as significantly altered levels. (B) Expression levels denoted in log counts per million (logCPM) of HER2 (ERBB2) amplified and copy number neutral samples in each cohort.
