# Supplementary material for: Early EGFR Inhibition Triggers Actionable Tumor and Microenvironmental Remodeling in Lung Cancer
Source: Cancer Res Commun. 2026 Aug 26;6(8):1993–2009. doi: 10.1158/2767-9764.CRC-26-0073 (PMC13510642; doi:10.1158/2767-9764.CRC-26-0073)
Supplement: Supplementary file 20 [file crc-26-0073_figure_s4_suppsf4.pdf]

Supp. Figure 4

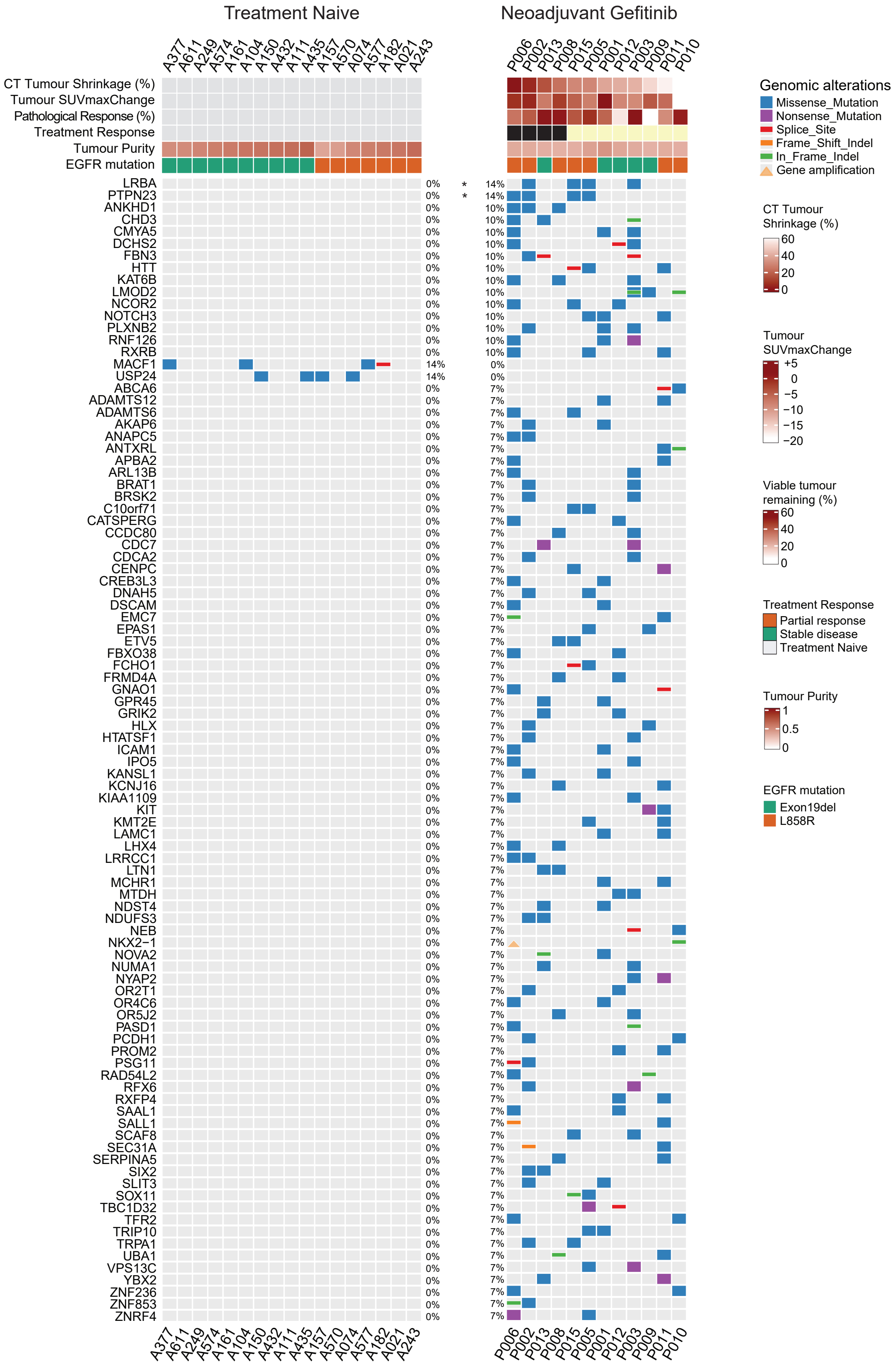

Supplementary Figure 4. Genomic landscape of 95 non-EGFR TKI resistance mutations found in neoadjuvant gefitinib treated *EGFR* mutated tumours. Significantly different mutation frequencies is denoted by asterisks (*: p<0.0001).
