# Supplementary material for: Early EGFR Inhibition Triggers Actionable Tumor and Microenvironmental Remodeling in Lung Cancer
Source: Cancer Res Commun. 2026 Aug 26;6(8):1993–2009. doi: 10.1158/2767-9764.CRC-26-0073 (PMC13510642; doi:10.1158/2767-9764.CRC-26-0073)
Supplement: Supplementary file 21 [file crc-26-0073_figure_s5_suppsf5.pdf]

Supp. Figure 5

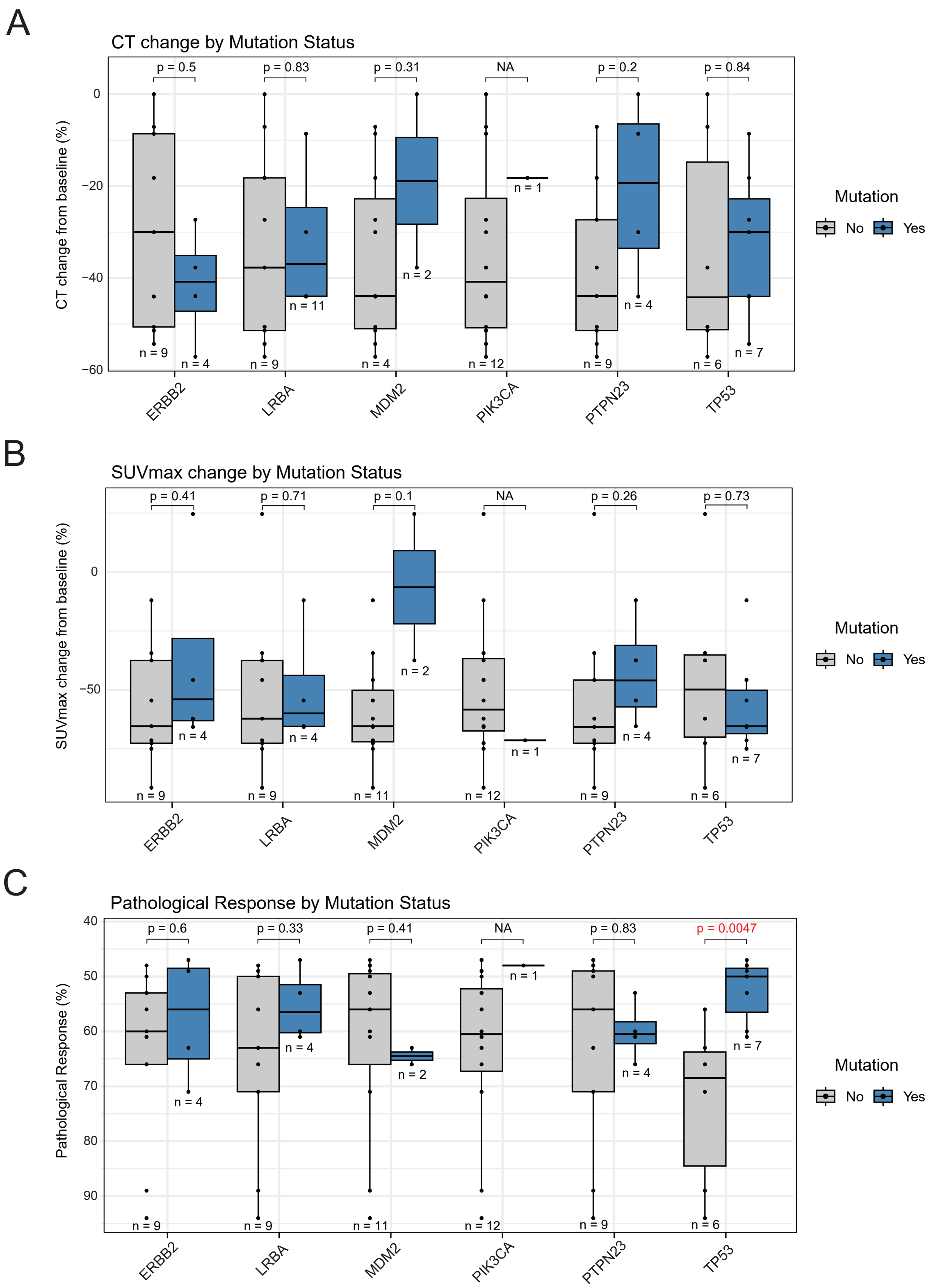

Supplementary Figure 5. Associations between mutations detected in neoadjuvant gefitinib treated *EGFR* mutated tumours and treatment response. (A) CT change from baseline (%), (B) SUVmax change from baseline (%), and (C) pathological response (%) stratified by mutation status in the genes *ERBB2, LRBA, MDM2, PIK3CA, PTPN23* or *TP53*. P-values are from Wilcoxon rank sum tests.
