# Supplementary material for: Early EGFR Inhibition Triggers Actionable Tumor and Microenvironmental Remodeling in Lung Cancer
Source: Cancer Res Commun. 2026 Aug 26;6(8):1993–2009. doi: 10.1158/2767-9764.CRC-26-0073 (PMC13510642; doi:10.1158/2767-9764.CRC-26-0073)
Supplement: Supplementary file 22 [file crc-26-0073_figure_s6_suppsf6.pdf]

Supp. Figure 6

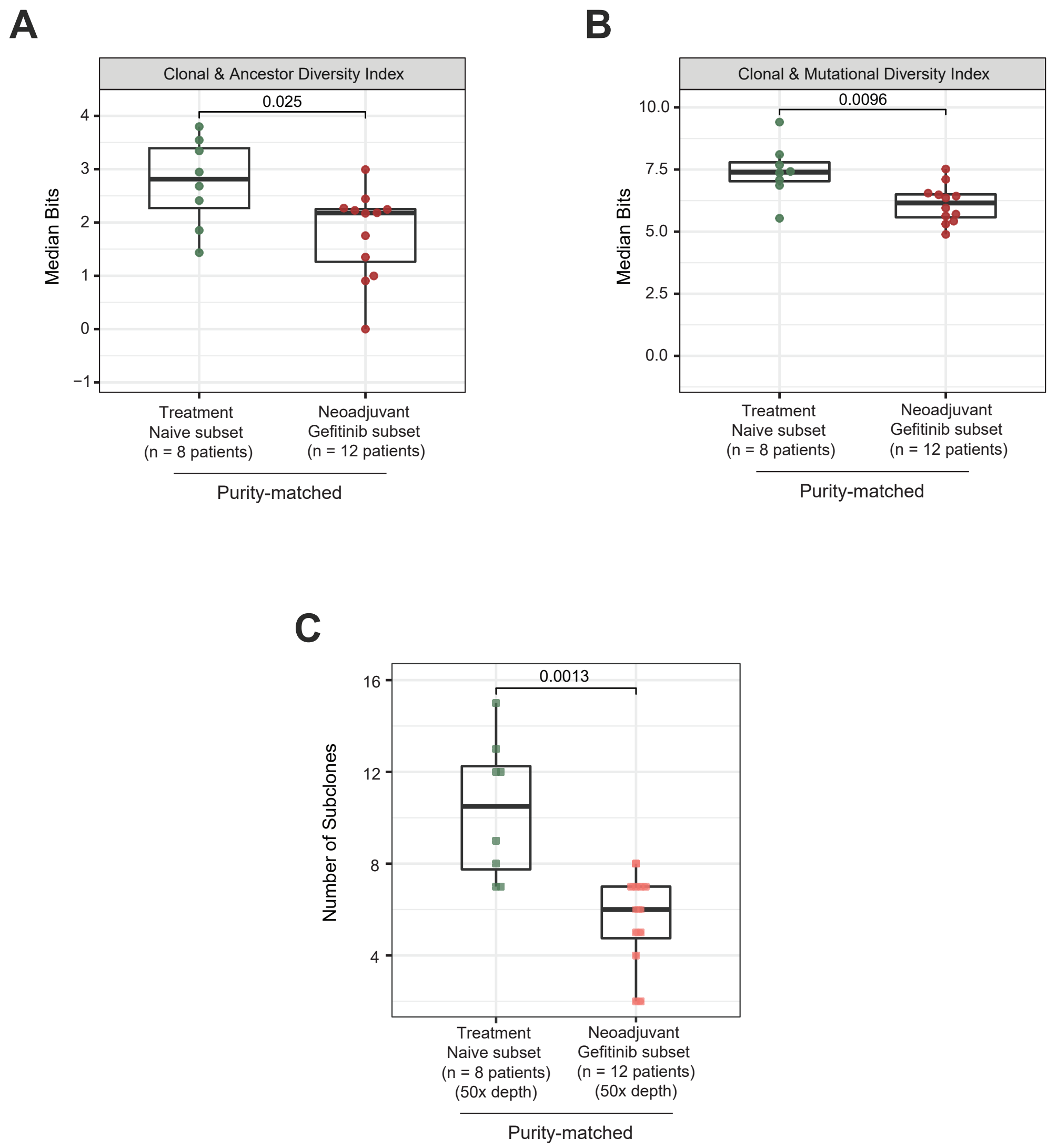

Supplementary Figure 6. Measures of clonal diversity in treatment naive and neoadjuvant gefitinib patients. (A-B) Two estimates for clone diversity in purity-matched cohorts, which consider phylogenetic structure (Clonal & Ancestor Diversity Index) and the mutational burden (Clonal & Mutational Diversity Index) for each patient. (C) Number of subclones detected for each patient tumour at matched depth and matched purity.
