# Supplementary material for: Early EGFR Inhibition Triggers Actionable Tumor and Microenvironmental Remodeling in Lung Cancer
Source: Cancer Res Commun. 2026 Aug 26;6(8):1993–2009. doi: 10.1158/2767-9764.CRC-26-0073 (PMC13510642; doi:10.1158/2767-9764.CRC-26-0073)
Supplement: Supplementary file 23 [file crc-26-0073_figure_s7_suppsf7.pdf]

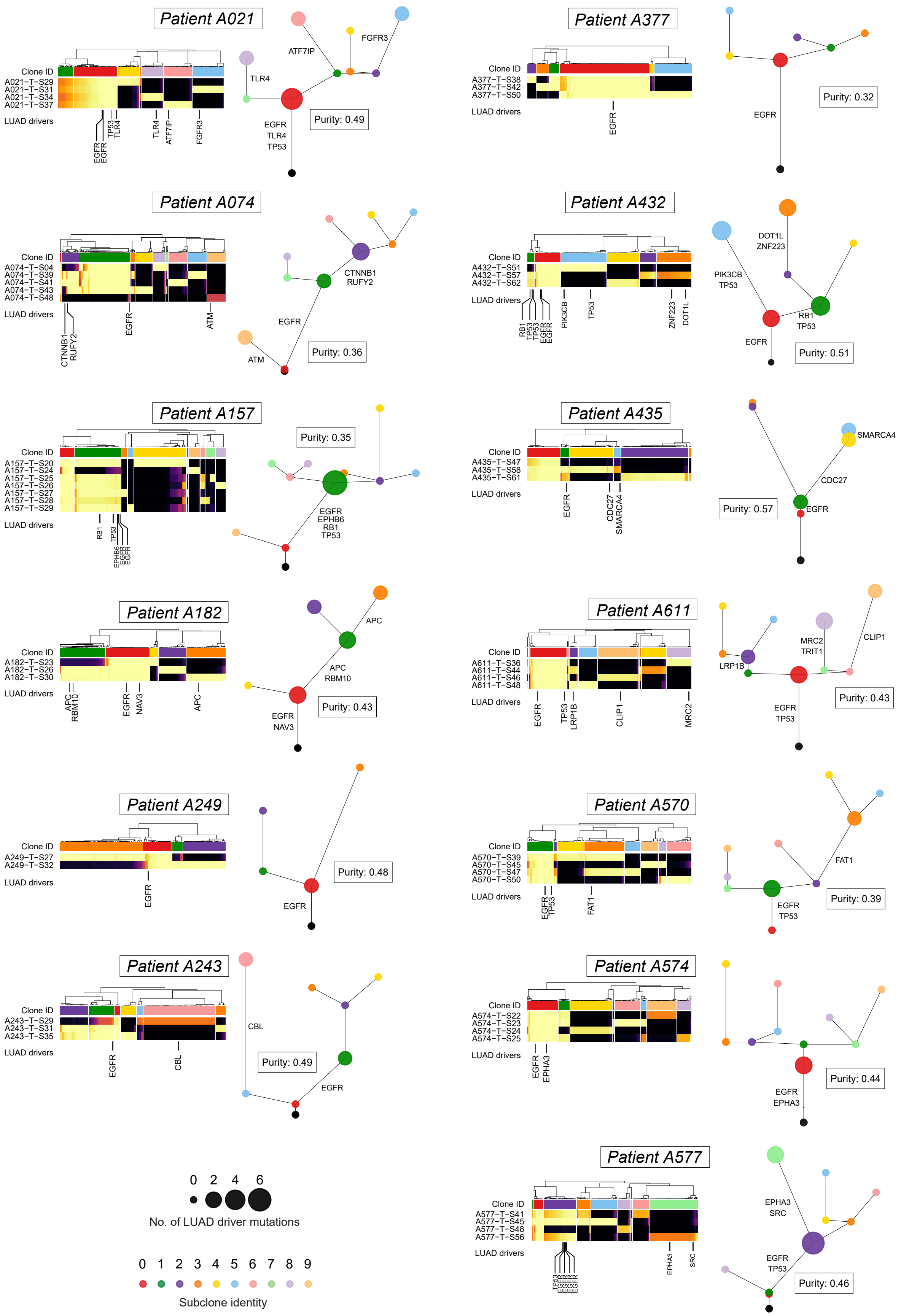

Supplementary Figure 7. Inferred clone phylogenies with tumour purity for treatment naive patients. Phylogenetic trees of detected clones from Treatment Naive patients, as inferred by CITUP. Only known LUAD (n=96) and Cancer Gene Census (n=359) driver genes are annotated for each clone on the tree. The mutation burden of a given clone is denoted by the vertical distance between itself and its parent node, whereas the horizontal distances between the nodes do not correspond to genetic distances.
