# Supplementary material for: Early EGFR Inhibition Triggers Actionable Tumor and Microenvironmental Remodeling in Lung Cancer
Source: Cancer Res Commun. 2026 Aug 26;6(8):1993–2009. doi: 10.1158/2767-9764.CRC-26-0073 (PMC13510642; doi:10.1158/2767-9764.CRC-26-0073)
Supplement: Supplementary file 25 [file crc-26-0073_figure_s9_suppsf9.pdf]

Supp. Figure 9

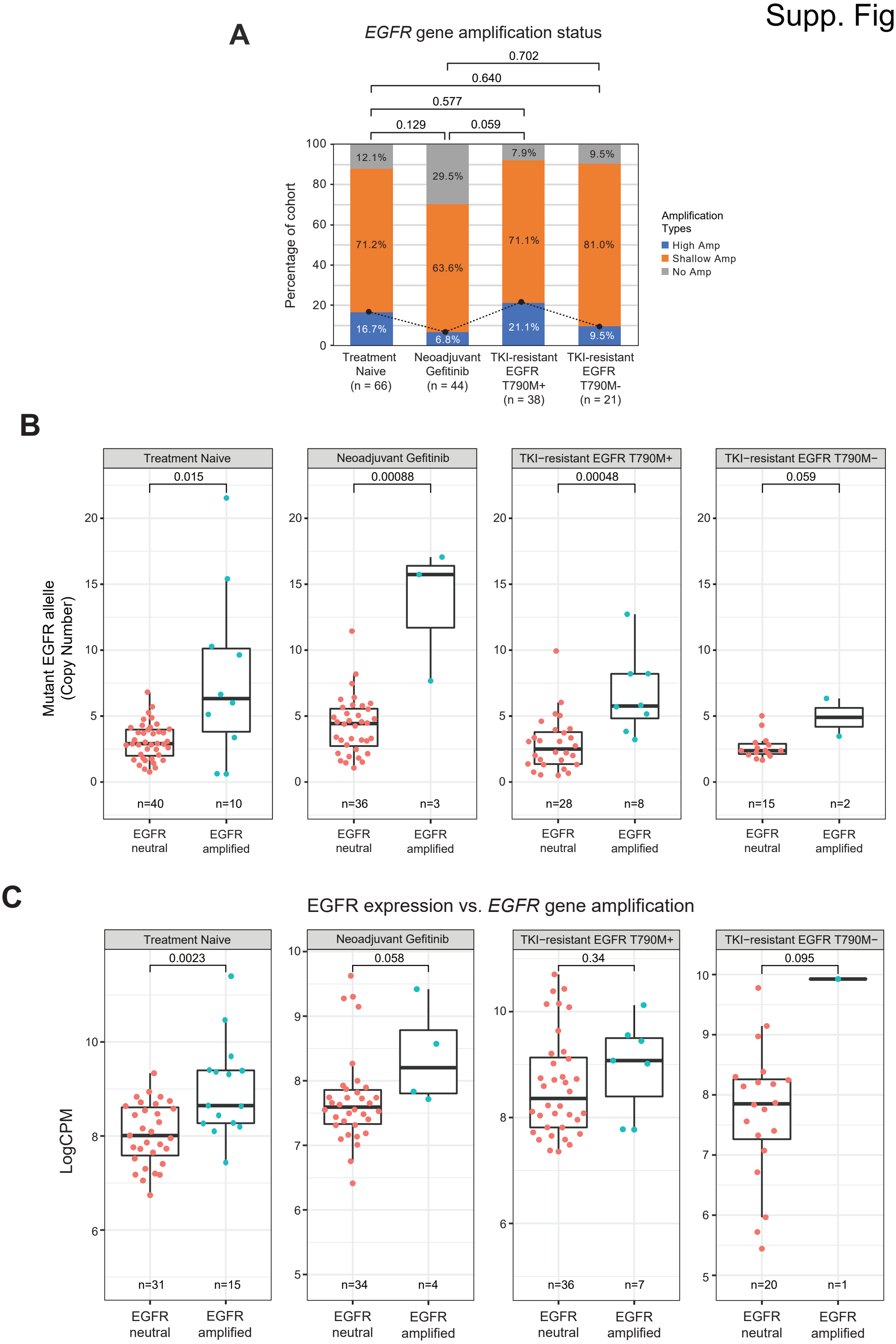

Supplementary Figure 9. EGFR gene amplification and expression levels. (A) Percentages of EGFR amplifications for each of the treatment naive, neoadjuvant gefitinib and TKI-Resistant cohorts as estimated by GISTIC2. P-values denote Chi-squared tests comparing the number of high amplification samples as a proportion of its cohort, against another cohort. (B) The number of EGFR mutant alleles estimated by EstimateClonality for EGFR amplified, and neutral samples. (C) Expression levels denoted in log counts per million (logCPM) of EGFR amplified and copy number neutral samples in each cohort.
