# Supplementary material for: Early EGFR Inhibition Triggers Actionable Tumor and Microenvironmental Remodeling in Lung Cancer
Source: Cancer Res Commun. 2026 Aug 26;6(8):1993–2009. doi: 10.1158/2767-9764.CRC-26-0073 (PMC13510642; doi:10.1158/2767-9764.CRC-26-0073)
Supplement: Supplementary file 26 [file crc-26-0073_supplementary_methods_suppsm.docx]

**Supplementary Methods**

*Somatic variant identification and CNVs identification*

WES data were aligned to the human reference genome (GRCh38) using BWA-MEM [(1)](https://paperpile.com/c/A0cNfs/8MwN). High confidence mutation calls were then determined using a Random-Forest methodology named SMuRF [(2)](https://paperpile.com/c/A0cNfs/w4f1); which is an ensemble method built upon five established variant callers FreeBayes [(3)](https://paperpile.com/c/A0cNfs/YQGw), Mutect2 [(4)](https://paperpile.com/c/A0cNfs/6pEu), Strelka2 [(5)](https://paperpile.com/c/A0cNfs/SZoP), VarScan [(6)](https://paperpile.com/c/A0cNfs/jxKr) and VarDict [(7)](https://paperpile.com/c/A0cNfs/0xdb). Copy numbers were estimated using the CNVKit [(8)](https://paperpile.com/c/A0cNfs/DX5w). To determine tumour cell fraction within the patient samples, five different purity estimators were used: PurBayes [(9)](https://paperpile.com/c/A0cNfs/hDEa), ABsCN-seq [(10)](https://paperpile.com/c/A0cNfs/780k), TitanCNA [(11)](https://paperpile.com/c/A0cNfs/I6ob), THetA2 [(12)](https://paperpile.com/c/A0cNfs/8H1P), and Sequenza [(13)](https://paperpile.com/c/A0cNfs/9JyE), of which the tumour purity estimate was determined using the average from these five methods.

*Tumour clonality and CNV analysis*

Estimation of the cancer cell fraction for each sample was carried out using estimateClonality tool [(14)](https://paperpile.com/c/A0cNfs/fSfH), using cut offs of 0.05 minimum variant read proportions, a minimum of 5 alternative reads, and a minimum depth of 30 reads for each variant. For mutational clustering of variants, Pyclone [(15)](https://paperpile.com/c/A0cNfs/Sz5l) was then run in iterative mode. To infer the phylogenetic trees, CITUP [(16)](https://paperpile.com/c/A0cNfs/h2c0) was used using the identified clusters from Pyclone, with the maximum number of clones set to 10. Singleton clusters were removed from input to CITUP. Manual review was carried out on the clustering results to restore known driver genes that were filtered out – a singleton cluster containing TP53 mutation was found to be present in all sectors, and thus added to the root of the patient clone tree (Supp. Table 8-9). To further measure clonal diversity taking into account mutational burden and phylogenetic structure, we used Pairtree (17) to infer clone phylogeny and calculate the respective Clonal & Mutation Diversity Index and the Clonal & Ancestor Diversity Index for each patient.

In some cases, the highest likelihood phylogenetic tree placed known *EGFR* driver mutations (L858R/Exon 19 indels) in non-root clones. We observe that the parental root clone in these patients had few mutations with only slightly higher predicted cancer cell fractions than *EGFR*. The *EGFR* drivers were also observed to have an estimated CCF higher than 70% percentile across all sectors of the patients. *EGFR* driver mutations are believed to be one of the earliest events in LUAD cancer progression and consequently are identified in the vast majority of cancer cells [(17)](https://paperpile.com/c/A0cNfs/RAXn). Subclonal *EGFR* mutations are extremely rare in LUAD, and cases of pseudo-heterogeneity in less sensitive assays could be attributed to heterogenous *EGFR* amplifications and signal dilution due to stromal cells [(18)](https://paperpile.com/c/A0cNfs/btza). *EGFR* driver mutations are also known to be significantly more truncal relative to mutations in non-driver genes which are more often branch mutations [(19)](https://paperpile.com/c/A0cNfs/6B0E). Consequently, due to generally low tumour purities, we adopted a conservative view of *EGFR* heterogeneity, and assume *EGFR* is usually a truncal mutation. For the clone trees in question, we place the *EGFR* driver in the root clone.

**References**

1.	[Li H. Aligning sequence reads, clone sequences and assembly contigs with BWA-MEM [Internet]. arXiv [q-bio.GN]. 2013. Available from:](http://paperpile.com/b/A0cNfs/8MwN) <http://arxiv.org/abs/1303.3997>

2.	[Huang W, Guo YA, Muthukumar K, Baruah P, Chang MM, Jacobsen Skanderup A. SMuRF: portable and accurate ensemble prediction of somatic mutations. Bioinformatics. 2019;35:3157–9.](http://paperpile.com/b/A0cNfs/w4f1)

3.	[Garrison E, Marth G. Haplotype-based variant detection from short-read sequencing [Internet]. arXiv [q-bio.GN]. 2012. Available from:](http://paperpile.com/b/A0cNfs/YQGw) <http://arxiv.org/abs/1207.3907>

4.	[Benjamin D, Sato T, Cibulskis K, Getz G, Stewart C, Lichtenstein L. Calling Somatic SNVs and Indels with Mutect2 [Internet]. bioRxiv. 2019 [cited 2022 May 6]. page 861054. Available from:](http://paperpile.com/b/A0cNfs/6pEu) <https://www.biorxiv.org/content/10.1101/861054v1>

5.	[Kim S, Scheffler K, Halpern AL, Bekritsky MA, Noh E, Källberg M, et al. Strelka2: fast and accurate calling of germline and somatic variants. Nat Methods. Nature Publishing Group; 2018;15:591–4.](http://paperpile.com/b/A0cNfs/SZoP)

6.	[Koboldt DC, Zhang Q, Larson DE, Shen D, McLellan MD, Lin L, et al. VarScan 2: somatic mutation and copy number alteration discovery in cancer by exome sequencing. Genome Res. 2012;22:568–76.](http://paperpile.com/b/A0cNfs/jxKr)

7.	[Lai Z, Markovets A, Ahdesmaki M, Chapman B, Hofmann O, McEwen R, et al. VarDict: a novel and versatile variant caller for next-generation sequencing in cancer research. Nucleic Acids Res. 2016;44:e108.](http://paperpile.com/b/A0cNfs/0xdb)

8.	[Talevich E, Shain AH, Botton T, Bastian BC. CNVkit: Genome-Wide Copy Number Detection and Visualization from Targeted DNA Sequencing. PLoS Comput Biol. 2016;12:e1004873.](http://paperpile.com/b/A0cNfs/DX5w)

9.	[Larson NB, Fridley BL. PurBayes: estimating tumor cellularity and subclonality in next-generation sequencing data. Bioinformatics. 2013;29:1888–9.](http://paperpile.com/b/A0cNfs/hDEa)

10.	[Bao L, Pu M, Messer K. AbsCN-seq: a statistical method to estimate tumor purity, ploidy and absolute copy numbers from next-generation sequencing data. Bioinformatics. 2014;30:1056–63.](http://paperpile.com/b/A0cNfs/780k)

11.	[Ha G, Roth A, Khattra J, Ho J, Yap D, Prentice LM, et al. TITAN: inference of copy number architectures in clonal cell populations from tumor whole-genome sequence data. Genome Res. 2014;24:1881–93.](http://paperpile.com/b/A0cNfs/I6ob)

12.	[Oesper L, Satas G, Raphael BJ. Quantifying tumor heterogeneity in whole-genome and whole-exome sequencing data. Bioinformatics. Oxford Academic; 2014;30:3532–40.](http://paperpile.com/b/A0cNfs/8H1P)

13.	[Favero F, Joshi T, Marquard AM, Birkbak NJ, Krzystanek M, Li Q, et al. Sequenza: allele-specific copy number and mutation profiles from tumor sequencing data. Ann Oncol. 2015;26:64–70.](http://paperpile.com/b/A0cNfs/9JyE)

14.	[McGranahan N, Favero F, de Bruin EC, Birkbak NJ, Szallasi Z, Swanton C. Clonal status of actionable driver events and the timing of mutational processes in cancer evolution. Sci Transl Med. 2015;7:283ra54.](http://paperpile.com/b/A0cNfs/fSfH)

15.	[Roth A, Khattra J, Yap D, Wan A, Laks E, Biele J, et al. PyClone: statistical inference of clonal population structure in cancer. Nat Methods. 2014;11:396–8.](http://paperpile.com/b/A0cNfs/Sz5l)

16.	[Malikic S, McPherson AW, Donmez N, Sahinalp CS. Clonality inference in multiple tumor samples using phylogeny. Bioinformatics. 2015;31:1349–56.](http://paperpile.com/b/A0cNfs/h2c0)

17.	[Passaro A, Malapelle U, Del Re M, Attili I, Russo A, Guerini-Rocco E, et al. Understanding EGFR heterogeneity in lung cancer. ESMO Open. 2020;5:e000919.](http://paperpile.com/b/A0cNfs/RAXn)

18.	[Yatabe Y, Matsuo K, Mitsudomi T. Heterogeneous distribution of EGFR mutations is extremely rare in lung adenocarcinoma. J Clin Oncol. 2011;29:2972–7.](http://paperpile.com/b/A0cNfs/btza)

19.	[de Bruin EC, McGranahan N, Mitter R, Salm M, Wedge DC, Yates L, et al. Spatial and temporal diversity in genomic instability processes defines lung cancer evolution. Science. 2014;346:251–6.](http://paperpile.com/b/A0cNfs/6B0E)
