# Supplementary material for: Early EGFR Inhibition Triggers Actionable Tumor and Microenvironmental Remodeling in Lung Cancer
Source: Cancer Res Commun. 2026 Aug 26;6(8):1993–2009. doi: 10.1158/2767-9764.CRC-26-0073 (PMC13510642; doi:10.1158/2767-9764.CRC-26-0073)
Supplement: Supplementary file 27 [file crc-26-0073_table_s10_suppst10.docx]

**Supplementary Table 10.** Representativeness of Study Participants

| **Cancer type** | ***EGFR* mutated non-small cell lung cancer (NSCLC)** |
| --- | --- |
| Considerations related to: | |
| Sex | *EGFR* mutated NSCLC is more common in women |
| Age | Most commonly diagnosed in middle-aged to older adults |
| Race/ethnicity | More prevalent in East Asian populations |
| Geography | Highest incidence in East Asia |
| Other considerations | Strongly associated with never- or light-smoking status |
| Overall representativeness of this study | This study was conducted in Singapore, and therefore represents an Asian population of study participants. The age (median 60 years, range 48-78 years), gender (54% female, 46% male) and ethnicity (n=13/13, 100% Asian) distribution would be considered representative of *EGFR* mutated NSCLC. |
