# Supplementary material for: Early EGFR Inhibition Triggers Actionable Tumor and Microenvironmental Remodeling in Lung Cancer
Source: Cancer Res Commun. 2026 Aug 26;6(8):1993–2009. doi: 10.1158/2767-9764.CRC-26-0073 (PMC13510642; doi:10.1158/2767-9764.CRC-26-0073)
Supplement: Supplementary file 28 [file crc-26-0073_table_s1_suppst1.docx]

**Supplementary Table 1.** Treatment-related adverse events

| ***n*=13 (All patients who received at least one dose of gefitinib)** | **Grade 1-2**  ***n* (%)** | **Grade 3-4**  ***n* (%)** |
| --- | --- | --- |
| Rash | 7 (54) | 0 |
| Diarrhoea | 4 (31) | 0 |
| Oral ulcers | 2 (15) | 0 |
| AST/ALT elevation | 0 | 1 (8) |
| Dry skin | 1 (8) | 0 |
| Nausea | 1 (8) | 0 |
