# Supplementary material for: Early EGFR Inhibition Triggers Actionable Tumor and Microenvironmental Remodeling in Lung Cancer
Source: Cancer Res Commun. 2026 Aug 26;6(8):1993–2009. doi: 10.1158/2767-9764.CRC-26-0073 (PMC13510642; doi:10.1158/2767-9764.CRC-26-0073)
Supplement: Supplementary file 29 [file crc-26-0073_table_s2_suppst2.docx]

**Supplementary Table 2.** Pre- and post-treatment primary tumour size and staging

| **Patient** | **Primary Tumour Size on CT at Diagnosis (cm)** | **Primary Tumour Size on CT Prior to Resection (cm)** | **Clinical Stage at Diagnosis**  **(AJCC 7^th^ edition)** | **Pathologic Stage at Resection  (AJCC 7^th^ edition)** |
| --- | --- | --- | --- | --- |
| P001 | 61 | 38 | T2N2M0 (3A) | T2N2M0 (3A) |
| P002 | 35 | 32 | T2aN1M0 (2A) | T1aN0M0 (1A)^*^ |
| P003 | 41 | 23 | T2aN1M0 (2A) | T1bN2M0 (3A) |
| P005 | 30 | 21 | T1bN2M0 (3A) | T1bN1M0 (2A) |
| P006 | 18 | 18 | T1bN0M0 (1A) | T1aN0M0 (1A) |
| P008 | 22 | 16 | T1bN0M0 (1A) | T1aN0M0 (1A) |
| P009 | 77 | 38 | T2N2M0 (3A) | T1aN0M0 (1A)^*^ |
| P010 | 35 | 16 | T2N2M0 (3A) | T2aN0M0 (1B)^*^ |
| P011 | 28 | 12 | T1bN2M0 (3A) | T1aN2M0 (3A) |
| P012 | 37 | 18 | T2aN2M0 (3A) | T2aN2M0 (3A) |
| P013 | 33 | 27 | T2N2M0 (2A) | T4N0M0 (3A)^*#^ |
| P014 | 28 | 26 | T3N0M0 (2B) | T1cN0M0 (1A) |
| P015 | 25 | 14 | T3N0M0 (2B) | T2aN0M0 (1B) |

^*^Four patients with nodal pCR

^#^One patient’s T stage upstaged due to intrapulmonary lymphovascular micrometastases
